# Whole Transcriptome Sequencing Reveals Cancer-Related, Prognostically Significant Transcripts and Tumor-Infiltrating Immunocytes in Mantle Cell Lymphoma

**DOI:** 10.3390/cells11213394

**Published:** 2022-10-27

**Authors:** Esra Esmeray Sönmez, Tevfik Hatipoğlu, Deniz Kurşun, Xiaozhou Hu, Burcu Akman, Hongling Yuan, Ayça Erşen Danyeli, İnci Alacacıoğlu, Sermin Özkal, Aybüke Olgun, Taner Kemal Erdağ, Hua You, Can Küçük

**Affiliations:** 1 İzmir International Biomedicine and Genome Institute, Dokuz Eylül University, İzmir 35340, Türkiye; 2 Basic and Translational Research Program, İzmir Biomedicine and Genome Center, İzmir 35340, Türkiye; 3 Department of Basic Oncology, Oncology Institute, Dokuz Eylül University, İzmir 35340, Türkiye; 4 Department of Pathology, Faculty of Medicine, Dokuz Eylül University, İzmir 35340, Türkiye; 5 Department of Pathology, Acıbadem University Altunizade Hospital, İstanbul 34662, Türkiye; 6 Department of Hematology, Faculty of Medicine, Dokuz Eylül University, İzmir 35340, Türkiye; 7 İzmir Tepecik Research and Training Hospital, Health Sciences University, İzmir 35020, Türkiye; 8 Department of Otorhinolaryngology, Faculty of Medicine, Dokuz Eylül University, İzmir 35340, Türkiye; 9 Chongqing Key Laboratory of Pediatrics, Ministry of Education Key Laboratory of Child Development and Disorders, Department of Hematology and Oncology, Children’s Hospital of Chongqing Medical University, Chongqing 401122, China; 10 International Science and Technology Cooperation Base of Child Development and Critical Disorders, National Clinical Research Center for Child Health and Disorders, Children’s Hospital of Chongqing Medical University, Chongqing 401122, China; 11 Department of Medical Biology, Faculty of Medicine, Dokuz Eylül University, İzmir 35340, Türkiye

**Keywords:** mantle cell lymphoma, WTS, mRNA, lncRNA, alternative transcript, oncogene, tumor suppressor, prognosis, tumor microenvironment

## Abstract

Mantle cell lymphoma (MCL) is an aggressive B-cell non-Hodgkin lymphoma (NHL) subtype characterized by overexpression of CCND1 and SOX11 genes. It is generally associated with clinically poor outcomes despite recent improvements in therapeutic approaches. The genes associated with the development and prognosis of MCL are still largely unknown. Through whole transcriptome sequencing (WTS), we identified mRNAs, lncRNAs, and alternative transcripts differentially expressed in MCL cases compared with reactive tonsil B-cell subsets. CCND1, VCAM1, and VWF mRNAs, as well as MIR100HG and ROR1-AS1 lncRNAs, were among the top 10 most significantly overexpressed, oncogenesis-related transcripts. Survival analyses with each of the top upregulated transcripts showed that MCL cases with high expression of VWF mRNA and low expression of FTX lncRNA were associated with poor overall survival. Similarly, high expression of MSTRG.153013.3, an overexpressed alternative transcript, was associated with shortened MCL survival. Known tumor suppressor candidates (e.g., PI3KIP1, UBXN) were significantly downregulated in MCL cases. Top differentially expressed protein-coding genes were enriched in signaling pathways related to invasion and metastasis. Survival analyses based on the abundance of tumor-infiltrating immunocytes estimated with CIBERSORTx showed that high ratios of CD8^+^ T-cells or resting NK cells and low ratios of eosinophils are associated with poor overall survival in diagnostic MCL cases. Integrative analysis of tumor-infiltrating CD8^+^ T-cell abundance and overexpressed oncogene candidates showed that MCL cases with high ratio CD8^+^ T-cells and low expression of FTX or PCA3 can potentially predict high-risk MCL patients. WTS results were cross-validated with qRT-PCR of selected transcripts as well as linear correlation analyses. In conclusion, expression levels of oncogenesis-associated transcripts and/or the ratios of microenvironmental immunocytes in MCL tumors may be used to improve prognostication, thereby leading to better patient management and outcomes.

## 1. Introduction

Mantle cell lymphoma (MCL) is an aggressive subtype of non-Hodgkin B-cell lymphoma with an incidence of 5–7% among all lymphomas [1]. MCL is genetically characterized by the t(11;14) (q13;q32) translocation, which leads to constitutive overexpression of the *CCND1* proto-oncogene due to the strong activity of the IgH enhancer juxtaposed near it, thereby leading to uncontrolled cell proliferation [2]. The majority of MCL cases are conventional MCLs (cMCLs) that are considered to originate from the neoplastic transformation of naive-like B-cells with no or a minimal number of *IGVH* mutations, whereas cases of the leukemic non-nodal MCL (nnMCL) subtype originate from memory-like B-cells with the *IGVH* gene mutated [3]. SOX11 transcription factor was reported to act as a proto-oncogene, which is overexpressed in all cMCLscorresponding to 90% of all MCLs [4].

MCL is an incurable malignancy despite recent advances in treatment options [5]. Standard therapeutic approaches such as immunochemotherapy and autologous stem cell transplantation may result in serious toxicity in MCL cases [6,7]. More importantly, these traditional therapeutic approaches are still not effective against most relapsed MCL patients [8]. Despite the presence of clinical heterogeneity in MCL that requires more personalized approaches, these types of therapies do not take into account the genetic or transcriptional alterations in tumors of individual MCL patients. Novel therapies targeting aberrant signaling pathways (e.g., BCR signaling) or biological processes (e.g., apoptosis) are under development [9]. However, targeted treatment approaches for MCL patients are still not adequate to account for individual differences in the biomolecular characteristics of the individual patients. Consequently, there is an urgent need for the identification of novel oncogenes or tumor suppressor genes which promote the development and malignant progression of MCLs in different MCL patient subgroups.

The prognosis of MCL may vary case-to-case; therefore, it is of utmost importance to identify patients bearing high risk in terms of prognosis and disease progression such that the most appropriate therapies can be applied with the correct timing. The MCL International Prognostic Index (MIPI), as well as the simplified MIPI risk score—which are based on the ECOG performance status, age, LDH levels, and white blood cell count—were established to identify high-, middle-, and low-risk group MCL patients [10]. The Ki67 proliferation index was reported to independently predict overall survival; hence, it is incorporated into MIPI, which is referred to as biological MIPI (MIPI-B) [11]. As these prognostic indices are not sufficient to account for the differences in the clinical outcome of MCL patients, researchers are seeking new prognostic biomarkers. As a part of this endeavor, a recent study proposed a circular RNA-based signature to predict good or inferior prognosis for MCL patients [12].

Several studies have previously revealed the genomic landscape of MCL tumors, which includes recurrent mutations in cancer-related genes such as *ATM*, *CCND1*, *TP53*, *NOTCH2*, or *RB1* [13,14,15,16]. Mutations of *KMT2D* and inactivation of *TP53* through deletions or mutations have recently been reported to be associated with MCL progression and short overall survival [17]. Unlike genomic investigations, only a few transcriptomic studies have been performed with MCL cases. In one of these studies, recurrent *NOTCH1* mutations were identified through analysis of whole transcriptome sequence data [18]. Another study proposed distinct molecular subsets of MCL identified through genomic and transcriptomic analyses [19]. Of significance, LINK-A lncRNA was detected with qRT-PCR to be overexpressed in the plasma of MCL patients, and suggested as a candidate oncogene based on in vitro functional experiments [20]. Furthermore, it has recently been suggested to cause ibrutinib resistance in MCL cell lines [21]. miRNA expression profiling of MCL tumors with a microarray platform identified miR-29 as a prognostic and pathogenic factor [22]. Among a few reports investigating the association between lncRNA expression and MCL patient prognosis, high expression of FOXP4-AS1 [23] or MALAT1 lncRNA [24] was shown to be associated with inferior prognosis. However, these transcriptional analyses in MCLs did not include a systematic investigation of the prognostic significance of mRNAs and lncRNAs upregulated in tumor cells as well as the immune cells in the tumor microenvironment (TME).

Previous reports on other cancer types including, but not limited to, non-small-cell lung cancer, breast cancer, and ovarian cancer showed that prognosis can be predicted by dichotomizing the patients based on transcript expression levels of mRNAs [25,26], lncRNAs [27,28], or alternative transcripts [29,30] in tumor tissues. The immune-cell composition in the tumor microenvironment is known to be associated with cancer patient prognosis [31,32]. With the development of the CIBERSORTx program, which identifies enriched immune cells within the tumor microenvironment using gene expression profile data [33], investigations that focus on the relationship between tumor microenvironment immune-cell ratios and cancer patient prognosis have sped up [34,35,36].

In this study, we first identified differentially expressed transcripts through whole transcriptome sequencing and revealed novel oncogene and tumor suppressor candidates. Furthermore, we performed survival analyses with mRNAs, lncRNAs, or alternative transcripts upregulated in MCL cases, as well as with tumor microenvironmental immunocytes enriched in MCL cases, to discover novel transcriptional and/or cellular biomarkers that can potentially be used during diagnosis for prognostication.

## 2. Materials and Methods

### 2.1. MCL Patient Information

Twenty-seven newly diagnosed and four relapsed MCL patients whose tumor biopsies were available at the Department of Medical Pathology at Dokuz Eylül University (DEU) Hospital between 2008–2017 were included in this study. All MCL cases were evaluated according to the WHO 2016 classification using morphological criteria and an appropriate immunohistochemical panel. All of the samples had a neoplastic lymphoid infiltrate composed of a monotonous population of small/medium sized cells with CD20, CD5, and cyclin D1 positive immunostaining. The tumor tissues were obtained during diagnosis for 25 of 27 diagnostic MCL cases and during relapse for the 4 relapsed MCL cases. A tumor biopsy of one of these MCL patients (Case-11) was obtained in November 2014. However, further diagnostic evaluations of this MCL patient were performed at a hospital other than DEU in a different city. In that hospital, the patient was diagnosed with MCL in November 2015. Another MCL patient was diagnosed with MCL in July 2013 at a center other than DEU. This patient was then followed up without any treatment until a tumor biopsy was obtained in January 2014 at the DEU Hospital. In addition to tumor tissue samples, the demographic, clinical, and pathological information of MCL patients available as DEU Hospital records were obtained for this study. Clinicopathologic and demographic information of 31 MCL patients are summarized in Table S1.

### 2.2. Total RNA Isolation from MCL Tumor Sections

Tissue sections were obtained by microtome from formalin-fixed paraffin-embedded (FFPE) tumor tissues from the Histopathology Core facility at IBG. At least 5 tumor sections, each of which being 8 μM thick, were prepared, and placed into 1.5 mL Eppendorf tubes. To minimize RNA degradation, tumor sections were stored at +4 °C until RNA isolation [37]. Total RNA isolations of MCL tumor sections were performed with the RNeasy FFPE kit (Qiagen Inc., Hilden, Germany) according to the manufacturer’s instructions.

### 2.3. FACS Sorting of Reactive Tonsil B-Cell Subtypes and RNA Isolations

#### 2.3.1. Preparation of Reactive Tonsil Cell Suspensions

Fresh tonsil tissues were obtained through routine tonsillectomy operations performed by the Department of Otorhinolaryngology at Dokuz Eylül University. Tonsil tissues were immediately placed into a container with 1X PBS (Gibco Life Technologies, Waltham, MA, USA) solution after the operation. Then, they were carried to the Department of Medical Pathology where half of the tonsil tissues were provided for diagnostic purposes. Tonsil tissues were homogenized by using forceps, and cell suspensions were prepared in 50 mL of 1X PBS/5 mM EDTA (Analiz Kimya, İzmir, Türkiye) solution. Tonsil cell suspensions were passed through 100 micron Falcon™ Cell Strainers (Corning, Glendale, AZ, USA) for the elimination of cell clumps and debris. After that, cell suspensions were incubated in the 1X ACK Lysing Buffer (ThermoFisher Scientific, Waltham, MA, USA) at a 1:4 ratio for 15 min. After centrifugation and washing with 1X PBS/5 mM EDTA solution, cells were suspended in 20 mL of 1X PBS/5 mM EDTA solution. Reactive tonsil cells were counted with the trypan blue (Sigma, St. Louis, MO, USA) exclusion assay.

#### 2.3.2. Immunostaining, FACS Sorting, and RNA Isolations

Thirty million cells were used for each reactive tonsil sample. Tonsil cells were stained with a cocktail of antibodies for CD77, CD38, CD23, and IgD, and incubated in ice for 20 min in a dark place. After that, cells were washed with 6 mL 1X PBS/5 mM EDTA solution and centrifuged at 400× *g* for 10 min. Cells were resuspended in 1 mL 1X PBS/5 mM EDTA after centrifugation. For dead cell labeling, 6 μL DAPI (BioLegend, San Diego, CA, USA) was added into tubes stained or unstained with the antibody cocktail. Each B-cell subtype was sorted with the FACS Aria III equipment at the IBG Flow Cytometry Unit into FACS tubes containing 1 mL RPMI 1640 (Gibco Life Technologies, Waltham, MA, USA). The sorted B-cell subtypes and their cell-surface antigen phenotype is as follows: Naive B-cells, IgD^+^/CD23^−^; centrocytes, IgD^−^/CD38^+^/CD77^−^; memory B-cells, CD38^−^/IgD^−^. The following antibodies were used during tonsil cell immunostainings: FITC mouse anti-human CD77 (BD Biosciences, San Jose, CA, USA), FITC anti-human CD77 (BioLegend, San Diego, CA, USA), PE mouse anti-human CD38 (BD Biosciences, San Jose, CA, USA), PE anti-human CD38 (BioLegend, San Diego, USA), PerCP-Cy™5.5 mouse anti-human CD23 (BD Biosciences, San Jose, CA, USA), PerCP/Cyanine5.5 anti-human CD23 (BioLegend, San Diego, CA, USA), APC mouse anti-human IgD (BD Biosciences, San Jose, CA, USA), and APC anti-human IgD (BioLegend, San Diego, CA, USA). FACS results were analyzed with the BD FACSDiva 8.0 software (BD Biosciences, San Jose, CA, USA). A representative FACS gating report is shown in Figure S1. B-cell subtypes were transferred to Eppendorf tubes post-sorting, followed by centrifugation at 500× *g* for 5 min. Supernatants were discarded, and cell pellets were resuspended in TRIzol reagent (ThermoFisher Scientific, Waltham, MA, USA). After that, total RNA samples were isolated with the RNeasy Mini Kit (Qiagen, Hilden, Germany) as per the manufacturer’s recommendations.

### 2.4. Whole Transcriptome Sequencing

Whole transcriptome sequencing of 10 MCL tumor RNA samples as well as 8 MCL tumor samples, one reactive tonsil naive B-cell sample, and one reactive tonsil centrocyte sample were performed, respectively, at Macrogen and Novogene as follows: An Agilent 2100 Bioanalyzer was used to evaluate the quantity and integrity of total RNA samples at Macrogen. A Qubit 2.0 Fluorometer (Life Technologies, Waltham, MA, USA), Agilent 2100 Bioanalyzer, and qPCR were used to evaluate the quantity and integrity of RNA samples delivered to Novogene for WTS. The RIN and DV200 scores of the total RNA samples sequenced are available in Table S2. The NGS libraries were prepared using Illumina TruSeq Stranded Total RNA with Ribo-Zero Human/Mouse/Rat Kit. First, ribosomal RNA biomolecules were removed from total RNA samples with the rRNA Removal Kit. Then, RNA was fragmented with a fragmentation buffer, and converted to cDNA with random hexamers as primers. Second-strand cDNA synthesis was performed by DNA Polymerase I in the presence of buffer solution, dNTPs, and RnazH. Having repaired ends of cDNAs, adenine nucleotides—and then, sequencing adaptors—were attached to cDNA fragments. After size selection and PCR enrichment steps, cDNA libraries were prepared. Paired-end 100 bp (Macrogene) or 150 bp (Novogene) NGS reads were obtained through sequencing with the Illumina HiSeq platform. Around 80 million total (i.e., 40 million paired-end) NGS reads were obtained per sequenced sample. Whole transcriptome sequencing of 17 total RNA samples (14 MCL cases, one naive B-cell [Control-02], one centrocyte [Control-05], and one memory B-cell [Control-03]) was performed at Novogene using the NEBNext Ultra Directional RNA Library Prep Kit according to the manufacturer’s recommendations, which follows the same procedure as the Illumina TruSeq Stranded Total RNA with Ribo-Zero Human/Mouse/Rat Kit. As the amounts of B-cell subset RNAs were, by themselves, not enough for WTS apart from Control-01 and Control-04, they were combined to obtain enough RNA samples. Control-02 is a combination of the total RNAs of naive B-cells isolated from the reactive tonsils of control cases 2, 3, 4, and 5. Control-03 sample is a combination of reactive tonsil memory B-cells of control cases 1, 6, and 7. Control-05 represented a combination of total RNA samples from centrocytes from control cases 3, 4, and 5 (Table S3).

### 2.5. Quantification of the Expressed Transcripts in MCL Tumor and Control Samples

#### 2.5.1. Quality Control of the Raw WTS Data

The initial quality control of the WTS data was performed by analyzing the basic NGS statistics (Table S4). Quality control (QC) for raw reads was performed by using the FASTQC tool (https://www.bioinformatics.babraham.ac.uk/projects/fastqc/, accessed on 21 July 2020). FASTQC tool provides a read-quality report in html format which includes scores such as k-mer score, per base sequence quality, per sequence quality, sequence duplication levels, and more.

#### 2.5.2. Mapping Reads to a Reference Genome

RNA-Seq reads were mapped to the current reference genome (UCSC GRCh38/Hg38) with HISAT2, a splice-aware mapping tool that applies the Burrows–Wheeler transform (BWT) and the Ferragina–Manzini (FM) indexing scheme [38].

#### 2.5.3. Counting Reads

After the alignment of the NGS reads to the reference genome, gene-expression levels in WTS data were estimated by generating a count matrix using the featureCounts tool [39]. “gencode.v29.gff3.gz” was used as the gene annotation reference file.

### 2.6. Principal Component Analysis

We assessed the transcriptomic similarities among the samples by performing sample-level QC through Principal Component Analysis (PCA) using the WTS data of the 32 tumor tissue as well as the 5 reactive tonsil B-cell subset samples. After obtaining count data by DESeq2 analysis, the data was transformed using the vst transformation algorithm, and the PCA object was created. PCA plots were produced by ggplot2 (version 3.3.6, Hadley Wickham, New York, NY, USA), ggfortify (version 0.4.14, Masaaki Horikoshi and Yuan Tang, https://CRAN.R-project.org/package=ggfortify; accessed on 15 October 2022) and ggrepel (version 0.9.1, Kamil Slowikowski, https://CRAN.R-project.org/package=ggrepel, accessed on 16 October 2022) libraries in RStudio.

### 2.7. Identification of the Differentially Expressed mRNAs, lncRNAs, and Alternative Transcripts

DESeq2 software [40] was used for the identification of differentially expressed mRNAs and lncRNAs (Figure S2A). A *p* value of less than 0.05, log2 fold change >2, and FDR (False Discovery Rate) <0.001 were considered as cutoffs for significantly DE genes. Differentially expressed alternative transcripts were identified as follows: Raw NGS reads were first aligned to the current human reference genome (Hg38) using the HISAT2 tool. Submodules in the StringTie program were used for the determination of alternative transcripts and calculating their amounts in the samples [41]. DESeq2 was used to identify alternative transcripts that were significantly differentially expressed between MCL tumors and the control group (Figure S2B).

### 2.8. Pathway Analysis of Differentially Expressed mRNAs, Alternative Transcripts, and lncRNAs

The Reactome Pathway Knowledgebase was used to identify the statistically significant pathways or biological processes associated with the top 100 mRNAs or top 100 alternative transcripts significantly differentially expressed in 32 tumor samples compared with the 5 reactive tonsil B-cell subset samples [42]. The bioinformatics workflow applied is, briefly, as follows: First, differentially expressed gene symbols were converted to Entrez gene IDs using the web-based geneID conversion program DAVID [43]. Then, the ReactomePA Bioconductor package (version 1.32.0, Guangchuang Yuab and Qing-Yu He, https://guangchuangyu.github.io/software/ReactomePA, accessed on 10 December 2022) in RStudio was used to identify the statistically significant pathways or biological processes associated with the top 100 mRNAs or top 100 alternative transcripts significantly differentially expressed in 32 tumor samples compared with the 5 reactive tonsil B-cell subset samples [44]. The adjusted *p*-value cutoff at 0.05 was chosen for the evaluation of statistical significance. The statistically significant pathways and biological processes associated with the significantly differentially expressed top 100 lncRNAs were determined by using the ncFANs program with default parameter settings [45].

### 2.9. Identification of the Candidate Oncogenes and Tumor Suppressor Genes

Candidate oncogenes were determined with the guidance of the differential expression data and the previously reported literature. Starting with the top significantly overexpressed mRNA or lncRNA determined with differential expression analysis of 32 MCL tumor samples and 5 control samples, each gene was searched in PubMed and Google Scholar using the combination of the following words or phrases: gene name and cancer. If a top significantly overexpressed mRNA or lncRNA was shown at least in one research article to have oncogenic activity in any type of cancer, the gene is considered to be an oncogene candidate. Ten tumor suppressor gene candidates were identified with the same workflow, except that the gene search started from the top significantly downregulated gene. If a top significantly down-expressed mRNA or lncRNA was shown at least in one research article to have tumor suppressor activity in any type of cancer, the gene is considered to be a tumor suppressor candidate. The PubMed and Google Scholar searches were performed in September 2020.

### 2.10. Microenvironmental Immunocyte Ratio Estimation via CIBERSORTx Analysis

CIBERSORTx software was used to estimate the immune-cell subset abundance in the tumor microenvironment of MCL tumor tissues based on the gene-expression profiles of MCL tumor samples and the LM22 gene signature matrix [33]. The LM22 gene signature matrix includes genes that distinguish 22 human hematopoietic cell phenotypes used to deconvolve immune-cell subsets. These immune cells are memory B-cells, naive B-cells, naive CD4^+^T-cells, CD8^+^ T-cells, activated memory CD4^+^ T-cells, resting memory CD4^+^ T-cells, Tfh, regulatory T-cells, gamma-delta T-cells, plasma cells, resting natural killer (NK) cells, activated NK cells, monocytes, M0 macrophages, M1 macrophages, M2 macrophages, resting mast cells, activated mast cells, resting dendritic cells, activated dendritic cells, eosinophils, and neutrophils. CIBERSORTx was run with the “Impute Cell Fractions Analysis” module to list the proportion of immune cells in bulk (FFPE) tissue samples using the WTS transcript expression data. A mixture file including the normalized gene-expression profiles of 31 MCL cases was created according to the CIBERSORTx input data format. “B-mode (bulk mode) Batch-correction” was enabled to minimize the impact of the cross-platform variation between signature matrix and mixture samples. The LM22 source GEP file was used for better batch-correction. Disable quantile normalization parameter was set as TRUE, which is recommended for RNA-Seq data, and the relative mode was used.

### 2.11. Immunohistochemistry and Hematoxylin–Eosin Staining

The sections from the FFPE tissues of the cases were stained with CD8 antibody (Anti-CD8 alpha antibody [EPR20305], ready-to-use, Abcam) for CD8^+^ T-cell detection, or with NCAM1 (Anti-NCAM1 antibody [EPR21827], ready-to-use, Abcam) antibody for NK cell detection using a Ventana Benchmark Ultra system according to the manufacturer’s instructions. CD8 or NCAM1 positivity and hematoxylin–eosin staining patterns were evaluated, and then photographed with an Olympus BX43 light microscope by an expert pathologist.

### 2.12. Survival Analysis

Survival analysis was performed to investigate the relationship between overexpressed transcripts, microenvironmental immunocytes, or clinicodemographic variables and MCL prognosis. Survival [46] and Survminer [47] R packages were used for survival analysis and Kaplan–Meier graphs. When dichotomizing the cases according to the expression levels of genes into two groups with high- and low-expressed cases, the median value of the expression levels of each gene was used as threshold value. The median values of TME cell ratios were set up as the thresholds to discriminate MCL patients with a high or low ratio of each of these cell types. The time between the diagnosis date and the event was used for overall survival analysis. Multivariate Cox’s regression analysis was performed in order to assess the independent prognostic impact of presumed oncogenes, different immune-cell compositions, stage of the disease, and initial treatment received. The “survival” R package was used for the multivariate Cox’s regression analysis. Transcripts and immune-cell compositions were binary variables with high and low values based on the median as the cutoff. For transcripts, low expression values—and for immune-cell composition, high ratio—were determined as the reference level. For R-CHOP treatment and for stages, stage 2 was taken as reference level. The hazard ratio of this analyses represents the patient death rate in this analysis. Three diagnostic MCL cases (Case-07, Case-11 and Case-19) were excluded from the Cox’s regression analyses as the patient data for the evaluated parameters were not complete for these MCL cases.

### 2.13. qRT-PCR

Reverse transcription of MCL tumor tissue samples and reactive tonsil B-cell subsets was performed using QuantiTect Reverse Transcription Kit (Qiagen, Germany) following the manufacturer’s recommendations. One to ten diluted cDNAs were used as templates for qPCR amplifications using QuantiTect SYBR Green qPCR Master Mix (Qiagen, Germany) and a 7500 Fast Real-Time PCR System (Applied Biosystems, Bedford, MA, USA). The specificities of the amplicons were evaluated by visualization of the melting curves generated using 7500 Software v2.3, and by running them in the 1X TAE gel if needed. Blanks were used for each primer pair and qPCR experiment to ensure a lack of contamination. Replicate samples were amplified, and the samples were excluded from quantification if at least one of the samples of the gene of interest or the housekeeping gene did not generate a specific amplicon. The ΔΔCt method was used for calculations of the relative levels of the *CCND1* mRNA and *SNHG5* lncRNA transcripts. The *RPS13* gene was used as the housekeeping gene for normalization of each evaluated gene. The nucleotide sequences of primer pairs used in this study is as follows: CCND1-qRT-PCR-F: 5′-GCCTCACACGCTTCCTCTC-3′; CCND1-qRT-PCR-R: 5′-CTGGCGCAGGCTTGACT-3′; SNHG5-qRT-PCR-F: 5′-TGTCTTCAGTGGCACAGT-3′; SNHG5-qRT-PCR-R: 5′-CCATTAAATATTCTCCCAGATGTTC-3′; RPS13-qRT-PCR-F: 5′-CGAAAGCATCTTGAGAGGAACAG-3′; RPS13-qRT-PCR-R: 5′-CGGTGAATCCGGCTCTCTATTA-3′.

### 2.14. Comparison of WTS and qRT-PCR Results with Linear Correlation Analysis

To evaluate the consistency between WTS and qRT-PCR expression values through calculation of the Pearson correlation coefficients, RStudio software version 4.0.2 (RStudio Team, Boston, MA, USA, https://www.rstudio.com/, accessed on 23 July 2022) was used, as described previously [48]. The WTS data values used in linear correlation analyses were initially obtained by dividing normalized counts per million (CPM) values of *CCND1* or *SNHG5* to the CPM values of the *RPS13* housekeeping gene for each sample after differential expression (DE) analysis of MCL tumor samples (*n* = 32) and reactive tonsil B-cell subset samples (*n* = 5). Then, the mean values of the B-cell subsets (two NBC, two CC, and one MBC) were calculated as the control group, followed by the normalization of each of the samples to this mean value. In correlation analysis, each B-cell subset was represented by a single value by taking the average values of each B-cell subset (i.e., NBC, CC, and MBC). Normalized values were log2 transformed for linear correlation analysis. Samples with CPM values lower than 1 for *CCND1* or *SNHG5* were excluded from the correlation analysis. Similar to WTS data, qRT-PCR expression levels of *CCND1* or *SNHG5* were normalized to those of *RPS13* with the ΔΔCt method, and two replicates of each sample were used in experiments. After that, the relative expression values of each control group and MCL tumor sample were normalized to the expression values of the mean of all available B-cell subset samples (two NBC, two MBC, and two CC). In correlation analysis, each B-cell subset was represented with their average values (i.e., NBC, CC, and MBC). At the final stage, log2-transformed relative expression values based on qRT-PCR were calculated and used as input for the linear correlation analyses. Samples with no amplification on either reference or target gene in any duplicates for all qRT-PCR results were excluded from the analysis. Moreover, samples with Ct values higher than 33 for the reference gene in qRT-PCR were filtered out.

### 2.15. Statistical Analyses

For differential transcript expression analyses with DESeq2, FDR-adjusted *p* values were obtained through correction of the Wald test *p* values with multiple testing by applying the Benjamini and Hochberg method [40]. The significance of the MCL survival differences between two patient groups dichotomized based on the expression of transcripts or immunocyte ratios was evaluated by calculating *p* values based on the log-rank test [48]. To evaluate the consistency between expression values obtained with whole transcriptome sequencing and qRT-PCR for selected transcripts, the *p* values were calculated via Pearson correlation testing for paired expressions [49].

## 3. Results

### 3.1. Overall Research and Analysis Plan of the Study

**Figure 1:**
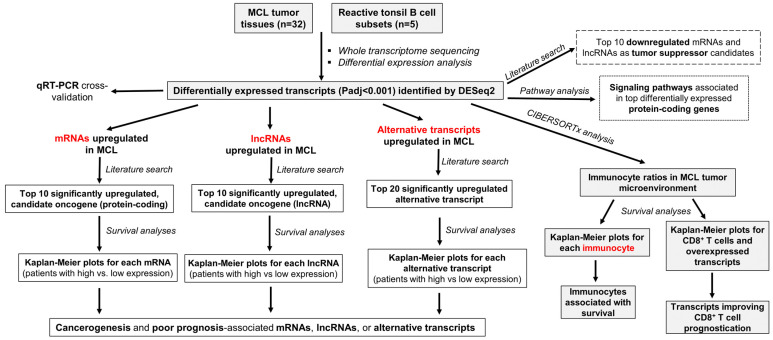
The overall workflow of the experiments and analyses.

The overall workflow of this study is shown in Figure 1, and includes the following steps: (1) Whole transcriptome sequencing (WTS) of 32 MCL tumor tissue samples as well as 5 reactive tonsil B-cell subtype samples; (2) Identification of differentially expressed mRNAs, lncRNAs, and alternative transcripts through computational bioinformatic analyses of the WTS data; (3) Investigation of the candidate oncogenes through a literature search of the top significantly overexpressed mRNAs and lncRNAs, with the assumption that if a gene is overexpressed in MCL cases, and implicated to be oncogenic in a different type of cancer, there is a high possibility that it can promote MCL development; (4) Determination of the tumor suppressor candidates downregulated in MCL tumor samples with the guidance of literature data; (5) Overall survival analyses by dichotomizing all or diagnostic MCL cases based on high or low expression for each of the candidate oncogene mRNA, lncRNA, or alternative transcripts to identify the most likely oncogene candidates associated with poor patent outcome; (6) CIBERSORTx analyses followed by MCL survival analyses based on the immunocyte ratios in the tumor microenvironment; (7) Identification of the higher-risk-group MCL cases by integrating poor prognosis-associated immunocyte ratio(s) and cancer-related overexpressed transcripts in survival analyses; (8) qRT-PCR analyses of selected transcripts for cross-validation of the WTS expression results, and linear correlation analysis to check sample-by-sample correlation of expression levels based on WTS and qRT-PCR measurements of selected transcripts.

### 3.2. CCND1, SOX11, ROR1-AS1, and LINK-A Are Overexpressed in MCL Tumors

**Figure 2:**
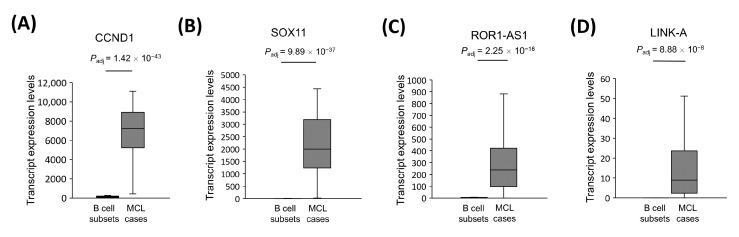
CCND1, SOX11, ROR1-AS1, and LINK-A transcripts are upregulated in MCL tumors. The transcript expression levels of CCND1 and SOX11 mRNAs as well as ROR1-AS1 and LINK-A lncRNAs in 32 MCL samples were compared with the 5 reactive tonsil B-cell subtype samples by DESeq2 analysis of whole transcriptome sequencing data. Box-and-whisker plots are shown for CCND1 (**A**), SOX11 (**B**), ROR1-AS1 (**C**), and LINK-A (LINC001139) (**D**) transcripts with the statistical significance indicated as adjusted *p* values over each plot.

To address whether our whole transcriptome analysis results are in agreement with the previously published literature, we evaluated the expression levels of CCND1 and SOX11 mRNAs as well as ROR1-AS1 and LINK-A (LINC001139) lncRNA expression levels in MCL tumor tissue samples. CCND1 and SOX11 mRNAs were significantly overexpressed in MCL cases compared with the reactive tonsil B-cell subsets (Figure 2A,B). Similarly, ROR1-AS1 and LINK-A lncRNA expression levels in MCL cases were much higher than those of control group samples (Figure 2C,D).

### 3.3. PCA Clustering of MCL and Control Samples by Transcriptome Profiles

**Figure 3:**
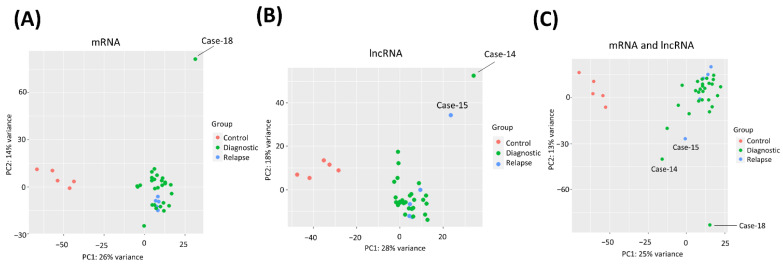
PCA plots of reactive tonsil B-cell subsets and MCL cases. PCA plots of diagnostic, relapsed MCL, and control samples generated based on only mRNA (**A**), only lncRNA (**B**), or both mRNA and lncRNA (**C**) transcript expression profiles are shown. Control samples represent reactive tonsil B-cell subset samples used for WTS. The patient codes of the outlier cases are indicated on the plots.

To evaluate the similarities and distances based on the transcriptomic profile among MCL tumor tissue and control samples, we performed PCA analysis. This analysis revealed that MCL cases and reactive tonsil B-cell subsets have distinct transcriptome profiles regardless of whether mRNA (Figure 3A), lncRNA (Figure 3B), or both mRNA and lncRNA (Figure 3C) transcriptome expression profiles were used. Of note, a diagnostic MCL sample (Case-18) was observed to be not present in the main MCL case cluster when mRNA expression profiles were used (Figure 3A). Interestingly, a diagnostic (i.e., Case-14) as well as a relapse (i.e., Case-15) MCL patient tumor sample were observed not to be inside the main cluster of diagnostic and relapse MCL cases based on the lncRNA transcriptome data (Figure 3B).

### 3.4. Differentially Expressed mRNAs, lncRNAs, and Alternative Transcripts in MCL Tumor Tissues

Transcript expression analyses of 32 MCL biopsy samples with DESeq2 revealed that 6644 mRNAs and 1067 lncRNAs are significantly (FDR < 0.001) upregulated in MCL cases compared with the reactive tonsil B-cell subset samples. In these MCL tumor samples, 2175 mRNAs and 989 lncRNAs were significantly downregulated. Next, we compared the alternative transcript expression levels between MCL tumor samples and reactive tonsil B-cell subsets, which revealed 10898 upregulated and 10643 downregulated alternative transcripts in MCL samples with statistical significance of FDR < 0.001.

### 3.5. Cancer-Related Signaling Pathways Are Enriched in Top Differentially Expressed Genes

**Figure 4:**
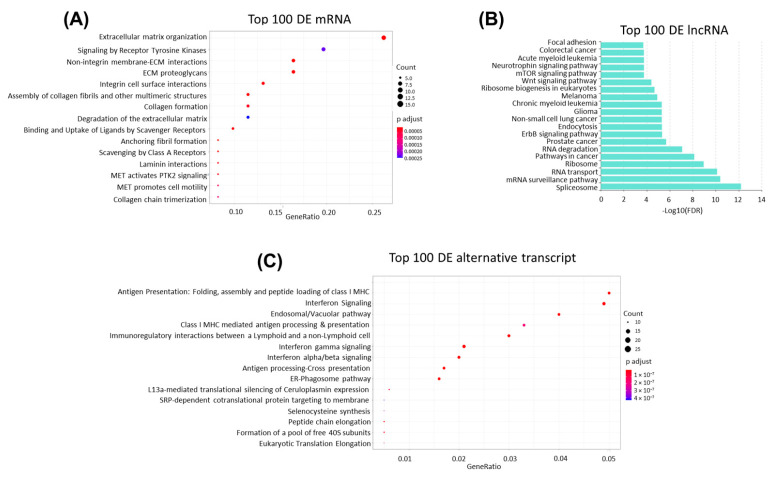
Biological processes or pathways associated with top 100 differentially expressed transcripts. Biological processes or pathways associated with statistically significant differentially expressed top 100 mRNAs (**A**), lncRNAs (**B**), and alternative transcripts (**C**) are shown with dot plots (**A**,**C**) or with a horizontal bar graph (**B**). GeneRatio and Count on plots represent the ratio of the genes that are annotated in a term and the number of genes that are a member to a given gene-set, respectively.

When the 100 protein-coding genes with the most significant differential expression in MCL tumor tissues were analyzed on the Reactome platform, we observed that many genes associated with invasion and metastasis are transcriptionally dysregulated in MCL tumors. The most prominent statistically significant signaling pathways associated with the first 100 genes included “non-integrin plasma membrane extracellular matrix (ECM) interactions”, “ECM proteoglycans”, and “ECM organization” (Figure 4A). Importantly, the most significantly differentially expressed genes were observed in “MET activates PTK2 signaling” and “MET promotes cell motility”, which are two of the invasion- and/or metastasis-related biological processes enriched in MCL cases. Top 100 differentially expressed lncRNAs were associated with pathways activated in cancer, including EGF receptor, Wnt, and mTOR signaling pathways (Figure 4B). When the top 100 alternative transcripts differentially expressed in MCL tumors were analyzed with the Reactome program, a total of 51 signaling pathways/biological processes were identified to be significantly (FDR < 0.001) enriched in MCL cases. Antigen processing and presentation, interferon signaling, and ER-phagosome pathways were among the most significant pathways associated with dysregulated alternative transcripts (Figure 4C).

### 3.6. The Most Significantly Overexpressed, Cancerogenesis-Associated Gene mRNAs in MCL Tumor Tissues

**Figure 5:**
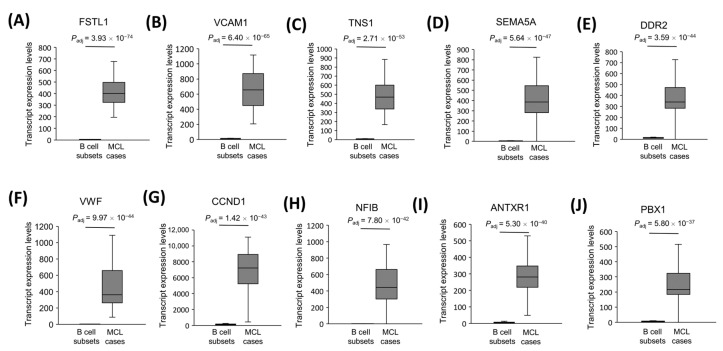
The transcript levels of top 10 cancerogenesis-associated protein-coding genes. Box-and-whisker plots of the 10 cancerogenesis-associated mRNAs that are most significantly overexpressed in 32 MCL samples compared with the 5 B-cell subtype samples are shown in the statistical significance order (**A**–**J**).

We assume that the protein-coding genes overexpressed in MCL cases are more likely to be associated with MCL cancerogenesis if these genes are implicated to be oncogenes in other cancer types. Based on the previously reported literature, we identified the top ten protein-coding genes that are the most significant overexpressed oncogene candidates in MCL tumor samples. We observed that FSTL1 (Figure 5A), VCAM1 (Figure 5B), TNS1 (Figure 5C), SEMA5A (Figure 5D), DDR2 (Figure 5E), VWF (Figure 5F), CCND1 (Figure 5G), NFIB (Figure 5H), ANTXR1 (Figure 5I), and PBX1 (Figure 5J) are the most significantly overexpressed genes that have been reported to be a potential oncogene in at least one cancer type. The literature information on the cancerogenic role of these mRNAs is shown in Table 1.

### 3.7. The Most Significantly Underexpressed Tumor Suppressor Candidate Gene mRNAs in MCL Tumor Tissues

**Figure 6:**
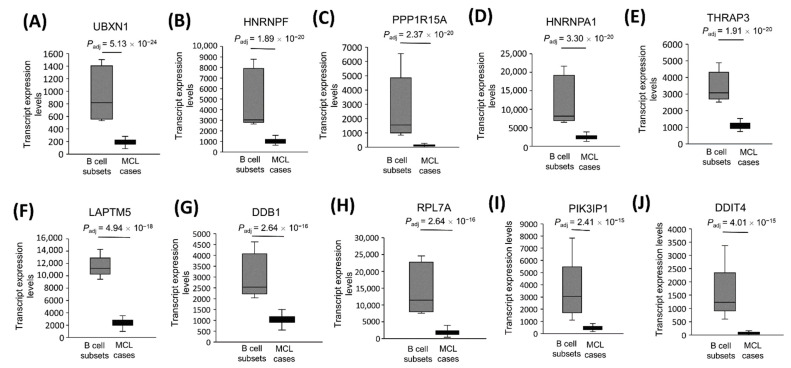
The transcript expression levels of the 10 most significantly downregulated tumor suppressor candidates in MCL. Tumor suppressor candidate transcript expression levels that are most significantly downregulated are shown for 32 MCL samples and 5 reactive tonsil B-cell subset samples as box-and-whisker plots in the statistical significance order (**A**–**J**). Adjusted *p* values show the statistical significance of expression differences between MCL and the control samples.

By following the same logic, we proposed that a protein-coding gene is more likely to be a tumor suppressor if it is implicated as a tumor suppressor gene in at least one type of cancer in the literature. To address this possibility, we performed a literature search for the most downregulated protein-coding genes, and identified those that are implicated as a tumor suppressor gene in at least one type of cancer (Table 2). Based on these criteria, we observed UBXN1 (Figure 6A), HNRNPF (Figure 6B), PPP1R15A (Figure 6C), HNRNPA1 (Figure 6D), THRAP3 (Figure 6E), LAPTM5 (Figure 6F), DDB1 (Figure 6G), RPL7A (Figure 6H), PIK3IP1 (Figure 6I), and DDIT4 (Figure 6J) as the most likely candidate tumor suppressor genes in mantle cell lymphoma.

### 3.8. The Most Significantly Overexpressed Oncogenesis-Associated lncRNA Genes

**Figure 7:**
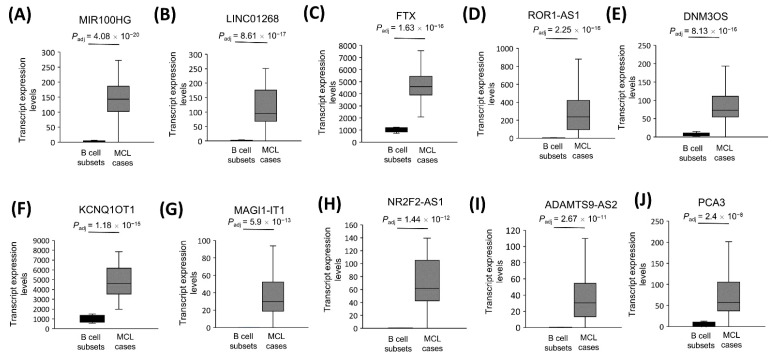
The transcript levels of top 10 cancerogenesis-associated lncRNA genes. Box-and-whisker plots of the 10 cancerogenesis-associated lncRNAs that are most significantly overexpressed in 32 MCL tumor samples compared with the 5 reactive tonsil B-cell subtype samples are shown in the order of decreasing statistical significance (**A**–**J**).

We also identified the top 10 overexpressed lncRNAs that have been implicated as oncogene candidates in at least one type of cancer (Table 3). This analysis showed significant upregulation of MIR100HG (Figure 7A), LINC01268 (Figure 7B), FTX (Figure 7C), ROR1-AS1 (Figure 7D), DNM3OS (Figure 7E), KCNQ1OT1 (Figure 7F), MAGI1-IT1 (Figure 7G), NR2F2-AS1 (Figure 7H), ADAMTS9-AS2 (Figure 7I), and PCA3 (Figure 7J) in 32 MCL tumor samples compared with the reactive tonsil B-cell subsets.

### 3.9. The Significantly Underexpressed Tumor Suppressor-Candidate Gene lncRNAs in MCL Tumor Tissues

**Figure 8:**
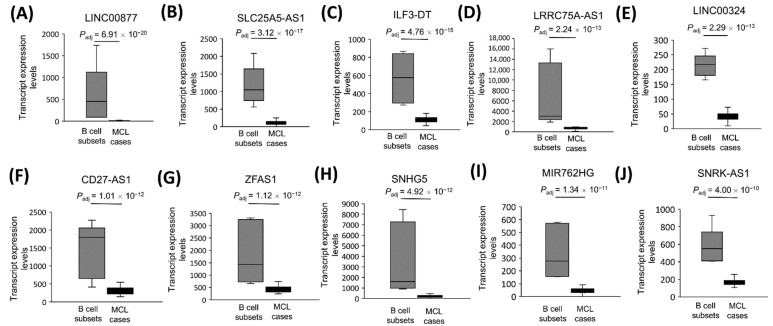
The transcript expression levels of the 10 most significantly downregulated tumor suppressor candidate lncRNA genes in MCL. Tumor suppressor candidate lncRNA expression levels that are most significantly downregulated are shown for 32 MCL samples and 5 reactive tonsil B-cell subset samples as box-and-whisker plots in the order of decreasing statistical significance (**A**–**J**). Adjusted *p* values show the statistical significance of expression differences between MCL and the control samples.

Similarly, we identified the top 10 most downregulated lncRNAs implicated as tumor suppressor genes in at least one type of cancer (Table 4). Based on this evaluation, LINC00877 (Figure 8A), SLC25A5-AS1 (Figure 8B), ILF3-DT (Figure 8C), LRRC75A-AS1 (Figure 8D), LINC00324 (Figure 8E), CD27-AS1 (Figure 8F), ZFAS1 (Figure 8G), SNHG5 (Figure 8H), MIR762HG (Figure 8I), and SNRK-AS1 (Figure 8J) were the top significantly downregulated tumor suppressor candidates in MCL cases.

### 3.10. The Relationship between Top Overexpressed Transcripts and MCL Survival

**Figure 9:**
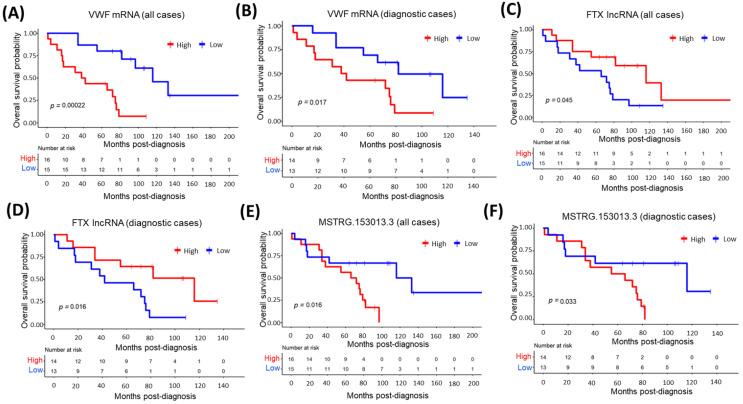
Top overexpressed transcripts significantly associated with MCL overall survival. MCL cases were dichotomized based on the mRNA or lncRNA expression levels of the top 10 cancerogenesis-related genes or the top 20 upregulated alternative transcripts. Kaplan–Meier curves of VWF mRNA (**A**,**B**), FTX lncRNA (**C**,**D**) and MSTRG.153013.3 alternative transcript (**E**,**F**) whose expression are significantly associated with poor or good overall survival in all (**A**,**C**,**E**) or diagnostic cases (**B**,**D**,**F**). *p* < 0.05 is considered statistically significant. High: High transcript expression. Low: Low transcript expression.

Next, we addressed whether transcript expression levels of the top 10 overexpressed oncogene candidates are associated with the overall survival of MCL patients. To address this question, we dichotomized all MCL patients (*n* = 31) or diagnostic MCL cases (*n* = 27) based on the transcript expression level of each of the top 10 cancerogenesis-associated protein-coding mRNA. Among these 10 protein-coding genes, VWF mRNA expression was significantly associated with inferior overall survival in all (Figure 9A) or diagnostic MCL cases (Figure 9B). Similarly, we performed overall survival analyses with the top overexpressed oncogenesis-related lncRNAs by dividing MCL cases based on transcript expression levels of each of these 10 lncRNA genes. In contrast to VWF mRNA, we observed that MCL cases with high expression of FTX lncRNA showed a significant association to overall survival, better than that of the low-expression group in all (Figure 9C) or diagnostic (Figure 9D) MCL cases. Next, we evaluated whether alternative transcripts can predict prognosis or not. To address this possibility, the top 20 overexpressed alternative transcripts were tested one-by-one for their ability to predict overall survival, which revealed a significant association between MSTRG.153013.3 transcript levels and the survival of MCL patients (Figure 9E,F).

### 3.11. The Relationship between Demographic or Clinical Variables and MCL Survival

We investigated the prognostic importance of certain demographic (i.e., age, gender) and clinical variables, as well as the MIPI (Mantle Cell Lymphoma International Prognostic Index), in diagnostic MCL cases. We observed that older age was significantly associated with poor overall survival (Figure S3A). However, no significant association with MCL survival was detected for other variables evaluated (Figure S3B–E).

### 3.12. CD8^+^ T-Cells in Tumor Microenvironment Predict Inferior MCL Survival

**Figure 10:**
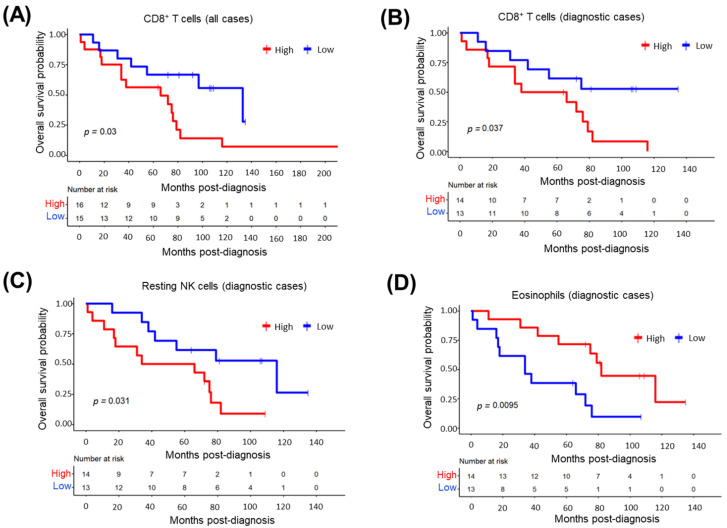
Microenvironmental immunocytes associated with overall survival of MCL patients. Kaplan-Meier plots show the tumor-infiltrating immunocytes that are associated with poor (**A**–**C**) or good (**D**) overall survival. High: High ratio of the immune cell. Low: Low ratio of the immune cell. All cases: 27 diagnostic and 4 relapsed MCL.

The tumor microenvironmental immunocyte composition of MCL cases was predicted using the CIBERSORTx program (Figure S4). To address whether there is any relationship between immunocytes infiltrating the tumor microenvironment and the overall survival of MCL patients, we divided all 31 patients or 27 diagnostic patients into two groups based on the presence of each immunocyte whose abundance in TME was predicted by the CIBERSORTx program. These analyses showed that MCL cases with a high proportion of CD8^+^T-cells have significantly poorer overall survival in all (Figure 10A) or diagnostic cases (Figure 10B). Resting NK-cell abundance in TME was associated with poor overall survival (Figure 10C), whereas eosinophils were associated with good survival (Figure 10D), in diagnostic MCL cases. No significant relationship with MCL survival was detected for all the other immunocytes evaluated. Tumor-infiltrating immune-cell abundances estimated with CIBERSORTx were cross-validated, either with immunohistochemistry (CD8^+^ T or NK cells) or with hematoxylin–eosin staining (eosinophils) in MCL cases representing high or low ratios of these cell types (Figure S5).

### 3.13. Impact of Tumor-Infiltrating CD8^+^ T-Cell Abundance and Cancer-Associated Transcripts on MCL Survival

**Figure 11:**
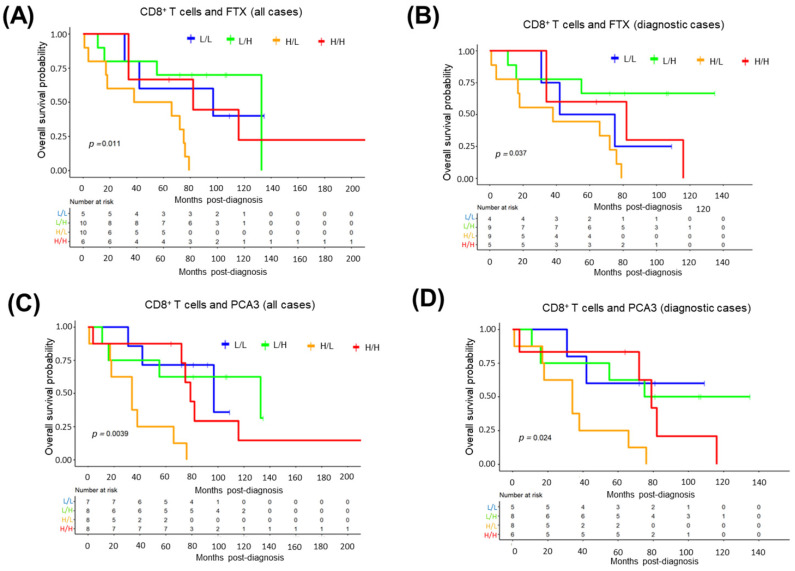
High tumor-infiltrating CD8^+^ T-cell ratio together with low FTX or PCA3 expression predicts high-risk MCL patients. Kaplan–Meier plots showing the curves based on a combination of tumor-infiltrating CD8^+^ T-cell ratios and FTX (**A**,**B**) or PCA3 (**C**,**D**) transcript expression levels. L/L: Low CD8^+^ T-cell abundance and low transcript expression; L/H: Low CD8^+^ T-cell abundance and high transcript expression; H/L: High CD8^+^ T-cell abundance and low transcript expression; H/H: High CD8^+^ T-cell abundance and high transcript expression.

As CD8^+^ T-cell abundance predicted inferior overall survival in all, as well as diagnostic, MCL cases, we performed MCL survival analysis by co-analyzing CD8^+^ T-cell ratios together with the top 10 cancer-associated, upregulated mRNAs or lncRNAs to evaluate the possibility of identifying high-risk MCL cases. When tumor-infiltrating CD8^+^ T-cell abundance was analyzed together with each of these transcripts, we observed that high CD8^+^ T-cells with low expression levels of FTX lncRNA predicted poorer overall survival compared to CD8^+^ T-cell ratio when all (Figure 11A) or diagnostic (Figure 11B) MCL cases were analyzed. Similar to these results, MCL cases with high CD8^+^ T-cell ratios and low PCA3 lncRNA levels predicted poor survival more significantly than evaluating CD8^+^ T-cells alone in all (Figure 11C) as well as diagnostic (Figure 11D) MCL cases. Of significance, MCL patients with a high ratio of CD8^+^ T-cells and high expression of CCND1 constituted a high-risk group among diagnostic MCL cases (Figure S6).

### 3.14. Multivariate Cox’s Regression Analysis Results of Overall Survival

Next, we evaluated the relationship between prognostically significant candidate oncogenes (i.e., VWF, FTX, or MSTRG.153013.3), tumor-infiltrating immunocytes (CD8^+^ T-cells, resting NK cells, or eosinophils), disease stage, treatment type, and overall patient survival by applying multivariate Cox’s regression analysis results in all (Figure S7A) or diagnostic (Figure S7B) MCL cases. These analyses showed that high VWF transcript expression in MCL cases was associated with poor overall survival when all MCL cases were evaluated (Figure S7A).

### 3.15. qRT-PCR Cross-Validated WTS Data of CCND1 and SNHG5 Transcripts

**Figure 12:**
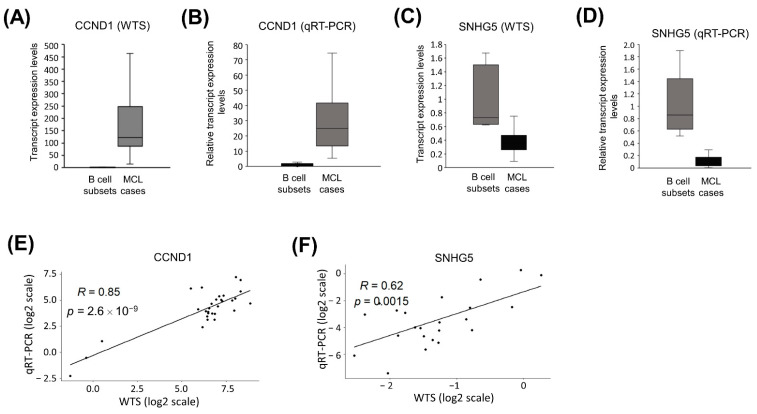
qRT-PCR cross-validates CCND1 and SNHG5 expression levels based on whole transcriptome sequencing. Box-and-whisker plots showing CCND1 mRNA WTS (**A**) and qRT-PCR (**B**) expression levels in MCL cases and control B-cell subsets. Box-and-whisker plots representing SNHG5 lncRNA WTS (**C**) and qRT-PCR (**D**). Linear correlation graphics comparing relative expression levels based on whole transcriptome sequencing and qRT-PCR for CCND1 mRNA (**E**) or SNHG5 lncRNA (**F**). CCND1 linear correlation graphic involves one value for each of NBC, MBC, and CC B-cell subsets as well as values for 24 MCL tumor tissue samples. SNHG5 linear correlation graphic involves the same set of control sample values in addition to 22 MCL tumor tissue samples. WTS: whole transcriptome sequencing; R: Pearson product-moment correlation.

As a proof-of-principle, we chose *CCND1* mRNA and *SNHG5* lncRNA for qRT-PCR validation of whole transcriptome sequencing results of MCL cases as well as control group samples (Table S3). Consistent with the whole transcriptome sequencing data that showed overexpression of *CCND1* transcripts in MCL tumor tissues (Figure 12A), we observed markedly higher expression of *CCND1* mRNA based on qRT-PCR (Figure 12B). *SNHG5* was one of the down-expressed genes in MCL tumor tissues based on WTS analysis (Figure 12C). qRT-PCR also showed downregulation of *SNHG5* lncRNA in MCL cases compared with reactive tonsil B-cell subsets (Figure 12D). To further evaluate the validity of transcript expression values determined with WTS, we performed linear correlation analyses by comparing the expression values of *CCND1* or *SNHG5* based on WTS or qRT-PCR. These analyses revealed that WTS and qRT-PCR expression values correlate significantly across MCL tumor samples and reactive tonsil B-cell subset samples for both *CCND1* (Figure 12E) and *SNHG5* (Figure 12F).

## 4. Discussion

Mantle cell lymphoma is an aggressive but clinically heterogenous B-cell non-Hodgkin lymphoma, of which limited knowledge exists on the role of transcripts and tumor microenvironment in patient prognostication. The discovery of novel transcriptional or cellular prognostic biomarkers may potentially improve, and/or provide alternative methods for, the current clinical evaluations to achieve better patient management, including—but not limited to—stratifying patients for more effective therapeutic interventions. To identify prognostically significant mRNAs, lncRNAs, and microenvironmental immunocytes, as well as to identify previously unknown MCL-associated genes in a comprehensive manner, we have applied WTS to a reasonably large cohort of mostly diagnostic MCL cases.

Most previous studies focused on genomic alterations in MCL cases [18,90]. Among a few reports on transcriptional alterations and pathogenesis of MCLs, certain cancer-related transcripts were identified. In a study involving a limited number of MCL cases, ROR1-AS1 was identified to be the most significantly overexpressed lncRNA compared to control samples. In vitro overexpression experiments revealed that ROR1-AS1 may promote cancerogenesis and decrease sensitivity to chemotherapy treatment [73]. Another study involving a few MCL cases, as well as MCL cell lines, reported lncRNAs associated with translation initiation complex by performing RNA immunoprecipitation (RIP)-seq [91]. Given the scarcity of transcriptomic studies focusing on the discovery of novel MCL-related genes, we hypothesized that upregulated or downregulated protein-coding transcripts and lncRNAs can act as oncogenes or tumor suppressor genes in MCL if they are also implicated in other cancer types. As expected, CCND1 was one of the top oncogene candidates identified in MCL tumors confirming previous studies for constitutive overexpression of CCND1 as an initial cancer-associated alteration that promotes uncontrolled proliferation [8]. Top underexpressed tumor suppressor candidates such as UBXN1, HNRNPA1, PPP1R15A, and LAPTM5 may also be contributing to tumor tissue formation through inhibition of apoptosis at initial stages of cancerogenesis (Table 2). Top differentially expressed alternative transcripts may be involved in evasion of tumor cells from CD8^+^T-cell attack through deregulation of antigen loading and presentation [9]. Interestingly, 7 of the top 10 oncogene candidate protein-coding genes identified (e.g., FSTL1, VCAM1, VWF) were shown in other cancer types to promote cell migration, invasion, or metastasis. Consistent with this finding, the top differentially expressed protein-coding genes enriched in MCL tumors were related either to the interaction of tumor cells with the extracellular matrix or promotion of invasion and metastasis, mainly through constitutive activation of MET signaling (Figure 4A). Transcriptional dysregulation of several metastasis-associated genes seems to play a critical role in dissemination of MCL tumor cells to distant sites such as bone marrow, liver, and spleen. Many of the top oncogene candidate lncRNAs (MIR100HG, LINC01268, FTX, etc.) were reported to promote cell growth or proliferation in different cancer types, suggesting that these lncRNAs may be involved at initial stages of tumorigenesis. However, many of these lncRNAs such as MAGI1-IT1 may have roles during invasion and metastatic dissemination.

The MCL International Prognostic Index (MIPI) is used for prognostication of MCL cases; however, the components of MIPI are not generally specific for cancer types. For example, high serum LDH levels may arise as a result of certain infections, anemia, or muscle trauma [92]. As older age is associated with poor survival of mantle cell lymphoma (Figure S3A), it was included as a component of the MIPI [10]. Interestingly, MIPI was not associated with MCL survival in our patient cohort, although older age was associated with inferior survival. This observation may be related to the lack of prognostic significance of other clinical variables that are components of MIPI, such as serum LDH levels (Figure S3D), in our MCL patients. RNA-Seq- or qRT-PCR-based quantification of prognostically significant transcripts (e.g., VWF or MRTG.153013.3) in MCL tumor tissues during routine clinical evaluations can potentially be used to improve prognostication of MCL cases during diagnosis. Consistent with the association of high VWF expression and shortened MCL survival, high expression of VWF may promote metastatic dissemination of the MCL tumor cells, as it was reported to promote metastasis in gastric adenocarcinoma [55] and breast cancer [93]. Many of the top overexpressed (e.g., FSTL1, TNS1, DDR2, or VWF) or underexpressed genes were suggested to promote invasion and/or metastasis in different cancer types. Genetic or epigenetic modulation of their expression may potentially prove effective against metastatic dissemination as long as these observations are reproduced in MCLs with in vitro or in vivo functional experiments. Alternatively, reconstitution of the expression of silenced tumor suppressor genes may inhibit cell proliferation and/or metastatic dissemination. Of note, preclinical studies showed that inhibition of VCAM1 may be an effective therapeutic option against pancreatic cancer [94]. Furthermore, inhibition of the PI3K pathway may be an effective strategy if the MCL cases with underexpression of PIK3IP1 have constitutive activation of the PI3K signaling pathway. Importantly, there are clinical trials evaluating the efficacy of PI3K pathway inhibitors in MCL [91], and certain side-effects associated with PI3K pathway inhibitors may be reduced by selecting an appropriate subset of patients through the evaluation of PI3KIP1 transcript expression in tumor samples of diagnostic MCL cases.

Accumulating evidence suggests that tumor-infiltrating lymphocytes can be associated with clinical outcomes for patients of different cancer types including, but not limited to, cervical cancer [95], osteosarcoma [96], and breast cancer [97]. The observation that CD8^+^ T-cells were associated with poor overall survival of MCL patients is consistent with previous reports, indicating a poor prognostic role for these immunocytes [98,99]. Given that MCL patients with abundant tumor-infiltrating CD8^+^ T-cells and low levels of FTX or PCA3 lncRNA can predict poorer overall survival (Figure 11), it may be possible to include WTS analysis during diagnosis to improve MCL prognostication. The observation that high CCND1 expression could identify a high-risk MCL group in the presence of infiltrating CD8^+^ T-cells (Figure S6) suggests that immunohistochemistry may potentially be applied for prognostication as long as these transcriptomic observations are reproduced at the protein level. Consistent with the reports on many other cancer types [100,101], the abundance of eosinophils was associated with better overall survival for diagnostic MCL patients. Unlike CD8^+^ T-cells, tumor-infiltrating resting NK cells or eosinophils were not significantly associated with MCL survival when relapsed MM cases were included. This observation may be related to differences in microenvironmental cell composition within the relapsed MCL tumor tissues. Evaluation of tumor-infiltrating immunocytes, together with clinical variables or prognostically significant transcripts, can be used to refine risk-groups during the diagnosis of MCL cases. However, it should be noted that our results in this study are largely exploratory, and they need to be validated in an independent cohort of MCL cases.

There are some limitations of this study. First, the number of MCL cases included in this study is not very large. Especially, the observations related to patient survival need to be validated in an independent cohort of MCL patients. Second, we have not compared the expression level changes in diagnostic vs. relapsed MCL cases, as the number of tumor samples from relapsed MCL cases was too few. Comparison of the changes in expression level in patient-matched tumor samples collected at diagnosis and relapse stages may provide useful information regarding oncogenes or tumor suppressors associated with MCL relapse. Third, our study has not evaluated the transcriptional biomarkers associated with drug resistance, as tumor samples were not collected after therapy. Future studies comparing transcript expression levels before and after specific types of therapies can potentially reveal transcriptional biomarkers whose expression predicts therapy response.

A recent study proposed four different prognostically distinct clusters based on integrative analysis of transcriptomic and genomic profiles [19] of MCL tumor samples. Given that Clusters 3 and 4 were identified as being associated with poor outcome, integration of the cancer-associated transcript (e.g., VWF) levels and/or TME immunocyte (e.g., CD8^+^T-cells) ratios into this model may be useful in further refinement of these MCL subgroups. It would also be important to evaluate the expression levels of the candidate oncogenes or tumor suppressors identified in this study in different parts of the tumor tissues in order to shed light onto their expression patterns and dynamics in different stages of malignant tumorigenesis, for which single-cell transcriptomic approaches may be especially useful. Of note, only one single-cell transcriptomic-based study has been reported for MCL tumors, which included a limited number of MCL cases [102].

Different quality checks were applied to ensure the reliability of the WTS analyses performed in this study, as total RNA from FFPE tumor tissues and freshly sorted tonsil B-cell subsets were analyzed altogether. First, we addressed whether mRNAs or lncRNAs known to be overexpressed in MCL cases have markedly high expression in our cohort of MCL cases or not. Consistent with the observations in previous reports [103], we detected significant upregulation of CCND1 and SOX11 mRNAs in mantle cell lymphoma cases (Figure 2). Similarly, ROR1-AS1 lncRNA was overexpressed in MCL cases compared to control cases in our cohort, which is in line with a previous publication showing ROR1-AS1 upregulation in MCL cases [73]. Overexpression of LINK-A lncRNA observed in MCL tumor samples further supported the reliability of the analytical pipeline for differential expression of WTS data applied in this study, as LINK-A was previously reported to be overexpressed in MCL cases [20]. As a second quality check for WTS analyses, overexpression of CCND1 and underexpression of SNHG5 were successfully cross-validated with qRT-PCR (Figure 12A–D). The finding that sample-by-sample comparison of CCND1 mRNA and SNHG5 lncRNA expression levels showed a strong positive correlation in linear correlation analyses further supported the reliability of the differential expression analyses of the WTS data of mantle cell lymphoma cases and reactive tonsil B-cell subsets. These observations suggest that transcript expression levels obtained from FFPE and fresh tumor samples can be analyzed together for the identification of differentially expressed transcripts when WTS involves ribosomal RNA depletion methods, as reported previously by another research group [104].

## 5. Conclusions

In conclusion, the oncogene and tumor suppressor gene candidates identified in MCL cases in this study may be involved in the development of MCL; however, future studies involving in vitro and/or in vivo experiments are needed to address this possibility. Importantly, we identified prognostically significant mRNAs and lncRNAs, as well as microenvironmental immunocytes. The transcripts, as well as immunocytes, identified through whole transcriptome sequencing of MCL tumors may potentially be applied in the clinic to improve the prognostication of MCL patients during diagnosis, thereby leading to better patient management and clinical outcomes.

## Figures and Tables

**Table 1:** The list of the top 10 overexpressed oncogene-candidate protein-coding transcripts in MCL cases.

Gene	Transcript Type	Associated Cancer Type	Cancer-Related Functions or Processes Promoted	Reference
FSTL1	mRNA	Colorectal cancer	Metastasis	Gu et al., 2018 [50]
VCAM1	mRNA	Breast cancer	Epithelial–mesenchymal transition (EMT)	Wang et al., 2014 [51]
TNS1	mRNA	Colorectal cancer	Proliferation and invasion	Zhou et al., 2018 [52]
SEMA5A	mRNA	Pancreatic cancer	Angiogenesis, proliferation, migration, anti-apoptosis	Sadanandam et al., 2010 [53]
DDR2	mRNA	Breast cancer	Metastasis, migration, invasion, EMT	Ren et al., 2014 [54]
VWF	mRNA	Gastric adenocarcinoma	Metastasis	Yang et al., 2018 [55]
CCND1	mRNA	Nasopharyngeal carcinoma	Cell cycle	Liu et al., 2012 [56]
NFIB	mRNA	Breast cancer	Cell survival	Liu et al., 2019 [57]
ANTXR1	mRNA	Glioma	Proliferation, migration, anti-apoptosis	Geng et al., 2019 [58]
PBX1	mRNA	Ovarian cancer	Proliferation	Park et al., 2008 [59]

**Table 2:** The list of top 10 underexpressed tumor suppressor candidate protein-coding transcripts in MCL cases.

Gene	Transcript Type	Associated Cancer Type	Cancer-Related Functions or Processes Regulated	Reference
UBXN1	mRNA	Osteosarcoma	Apoptosis	Wang et al., 2015 [60]
HNRNPF	mRNA	Breast cancer	EMT * suppression	Huang et al., 2017 [61]
PPP1R15A	mRNA	Burkitt’s lymphoma	Apoptosis	Hollander et al., 2001 [62]
HNRNPA1	mRNA	Ovarian cancer	Proliferation, motility, angiogenesis, and apoptosis	Rodriguez-Aguayo et al., 2017 [63]
THRAP3	mRNA	Cervical cancer	DNA damage response	Beli et al., 2012 [64]
LAPTM5	mRNA	Multiple myeloma	Cellular differentiation, apoptosis	Hayami et al., 2003 [65]
DDB1	mRNA	HPV-associated cancers	Cellular senescence	Kotake et al., 2009 [66]
RPL7A	mRNA	Osteosarcoma	Cell growth, differentiation	Zheng et al., 2009 [67]
PIK3IP1	mRNA	Hepatocellular carcinoma	Proliferation, motility	He et al., 2008 [68]
DDIT4	mRNA	Breast cancer	Proliferation, cell growth	DeYoung et al., 2008 [69]

* Epithelial–mesenchymal transition.

**Table 3:** The list of the top 10 overexpressed oncogene-candidate lncRNAs in MCL cases.

Gene	Transcript Type	Associated Cancer Type	Cancer-Related Functions or Processes Promoted	Reference
MIR100HG	lncRNA	Laryngeal squamous cell carcinoma	Proliferation, migration, invasion	Huang et al., 2019 [70]
LINC01268	lncRNA	Acute myeloid leukemia	Cell growth, anti-apoptosis	Chen et al.,2020 [71]
FTX	lncRNA	Gastric cancer	Proliferation, migration, invasion	Li et al., 2019 [72]
ROR1-AS1	lncRNA	Mantle cell lymphoma	Cell growth	Hu et al., 2017 [73]
DNM3OS	lncRNA	Gastric cancer	Proliferation, migration, invasion, EMT *	Wang et al., 2019 [74]
KCNQ1OT1	lncRNA	Non-small-cell lung cancer	Proliferation, anti-apoptosis	Kang et al., 2019 [75]
MAGI1-IT1	lncRNA	Epithelial ovarian cancer	Invasion, metastasis	Gao et al., 2019 [76]
NR2F2-AS1	lncRNA	Nasopharyngeal carcinoma	Proliferation, anti-apoptosis	Qin and Qin, 2020 [77]
ADAMTS9-AS2	lncRNA	Tongue squamous cell carcinoma	Proliferation, migration, EMT *	Li et al., 2019 [78]
PCA3	lncRNA	Prostate cancer	Proliferation, migration, invasion, anti-apoptosis	Zhang et al., 2019 [79]

* Epithelial–mesenchymal transition

**Table 4:** The list of the top 10 underexpressed tumor suppressor candidate lncRNAs in MCL cases.

Gene	Transcript Type	Associated Cancer Type	Cancer-Related Functions or Processes Regulated	Reference
LINC00877	lncRNA	Pheochromocytomas and paragangliomas	Metastasis	Ghosal et al., 2022 [80]
SLC25A5-AS1	lncRNA	Gastric cancer	Cell growth, apoptosis	Li et al., 2019 [81]
ILF3-DT	lncRNA	Cervical cancer	Autophagy	Feng et al., 2021 [82]
LRRC75A-AS1	lncRNA	Colorectal cancer	Proliferation, migration	Chen et al., 2019 [83]
LINC00324	lncRNA	Breast cancer	Proliferation, invasion, migration, apoptosis	Wang et al., 2020 [84]
CD27-AS1	lncRNA	Acute myeloid leukemia	Proliferation, cellular senescence, apoptosis	Tao et al., 2021 [85]
ZFAS1	lncRNA	Breast cancer	Proliferation, migration, invasion	Fan et al., 2018 [86]
SNHG5	lncRNA	Gastric cancer	Proliferation, metastasis	Zhao et al., 2016 [87]
MIR762HG	lncRNA	Ovarian cancer	Downregulated in ovarian cancer	Wang et al., 2019 [88]
SNRK-AS1	lncRNA	Hepatocellular carcinoma	Downregulated in hepatocellular carcinoma	Zhang et al., 2021 [89]

## Data Availability

The raw data files of whole transcriptome sequencing were uploaded to the NCBI SRA repository and can be accessed through the following link: https://dataview.ncbi.nlm.nih.gov/object/PRJNA882247?reviewer=8eeg2hfb350ihnpm1t12ahjcoj (accessed on 20 September 2022).

## Supplementary Materials

The following supporting information can be downloaded at: https://www.mdpi.com/article/10.3390/cells11213394/s1, Figure S1: FACS gatings during isolation of B cell subtypes from a representative reactive tonsil sample; Figure S2: Computational bioinformatic analyses workflows of the whole transcriptome sequencing data; Figure S3: The relationship between clinicopathological, demographic variables and MCL survival; Figure S4: The microenvironmental immunocyte composition of MCL cases; Figure S5: IHC or HE stainings confirm CIBERSORTx estimates in MCL cases; Figure S6: High risk MCL patients with a high ratio of CD8^+^ T cells and high CCND1 transcript expression levels; Figure S7: Forest plots of multivariate Cox’s regression analysis for overall survival; Table S1: The summary of demographic and clinicopathological characteristics of the MCL cases; Table S2: RIN and DV200 scores of MCL and control samples; Table S3: Control group samples used in WTS and qRT-PCR validation; Table S4: Basic statistics of whole transcriptome sequencing.
