# Supplementary material for: Whole Transcriptome Sequencing Reveals Cancer-Related, Prognostically Significant Transcripts and Tumor-Infiltrating Immunocytes in Mantle Cell Lymphoma
Source: Cells. 2022 Oct 27;11(21):3394. doi: 10.3390/cells11213394 (PMC9654955; doi:10.3390/cells11213394)
Supplement: Supplementary file 1 [file cells-11-03394-s001.zip › 28.10.2022-cells-1956495-SI Figures-MCL WTS-MDPI_Cells.pdf]

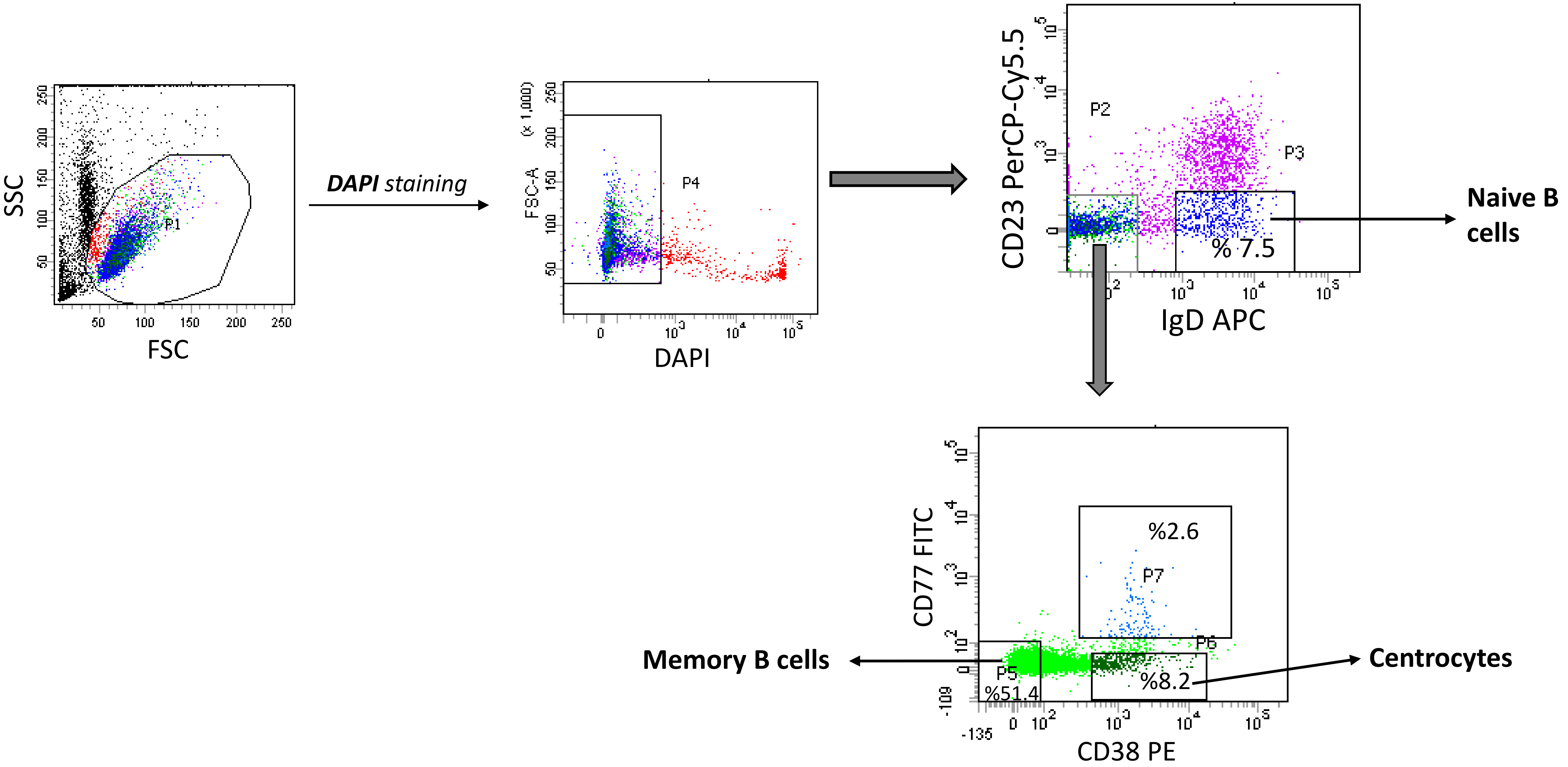

Figure S1. FACS gatings during isolation of B cell subtypes from a representative reactive tonsil sample. Flow cytometry analyses were performed with BD FACS Diva 7.0. DAPI staining was used to label and eliminate dead tonsil cells.

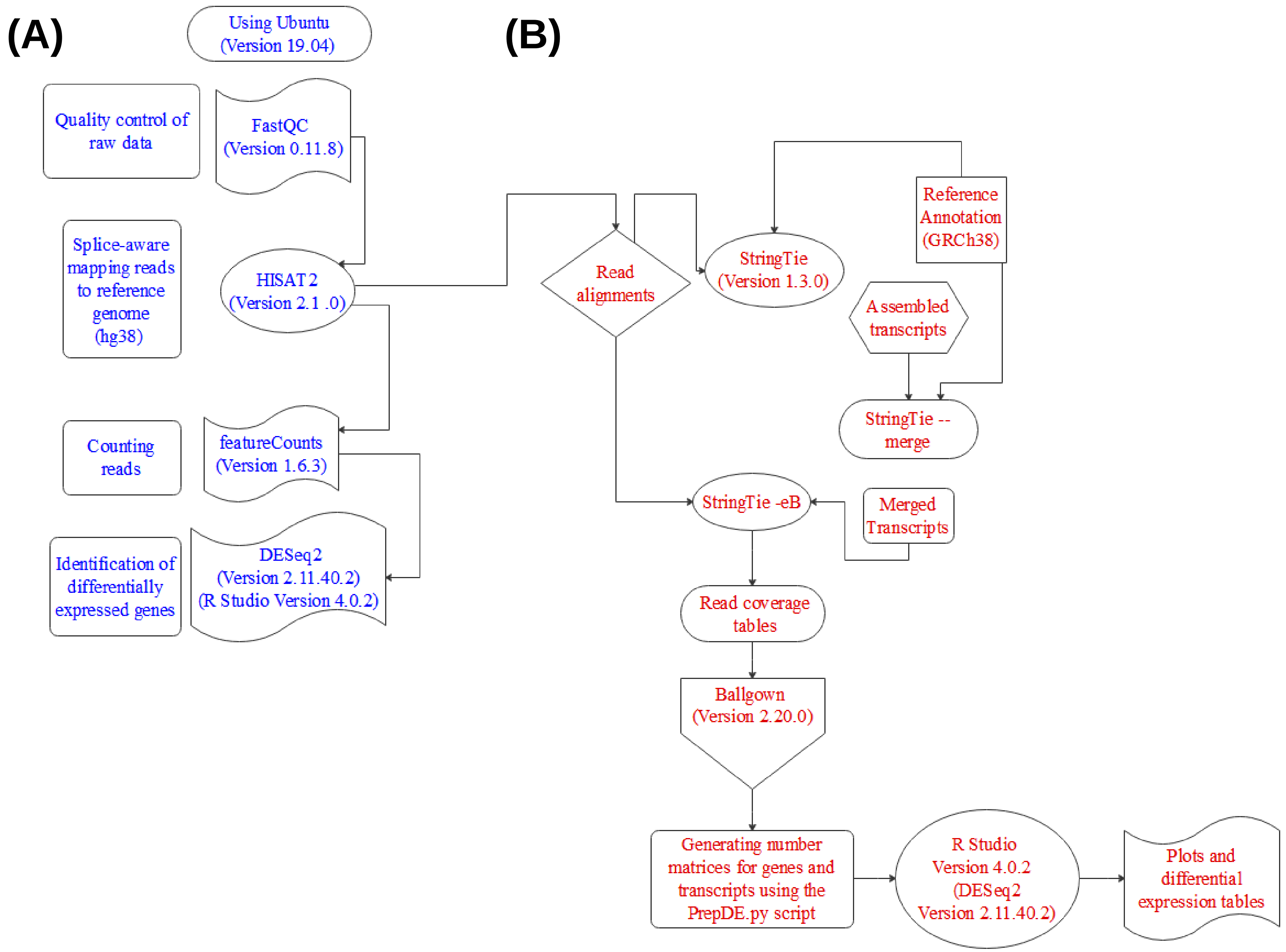

Figure S2. Computational bioinformatic analyses workflows of the whole transcriptome sequencing data. **A)** Differential expression analysis pipeline for mRNA and lncRNA. **B)** Differential expression analysis pipeline for alternative transcripts.

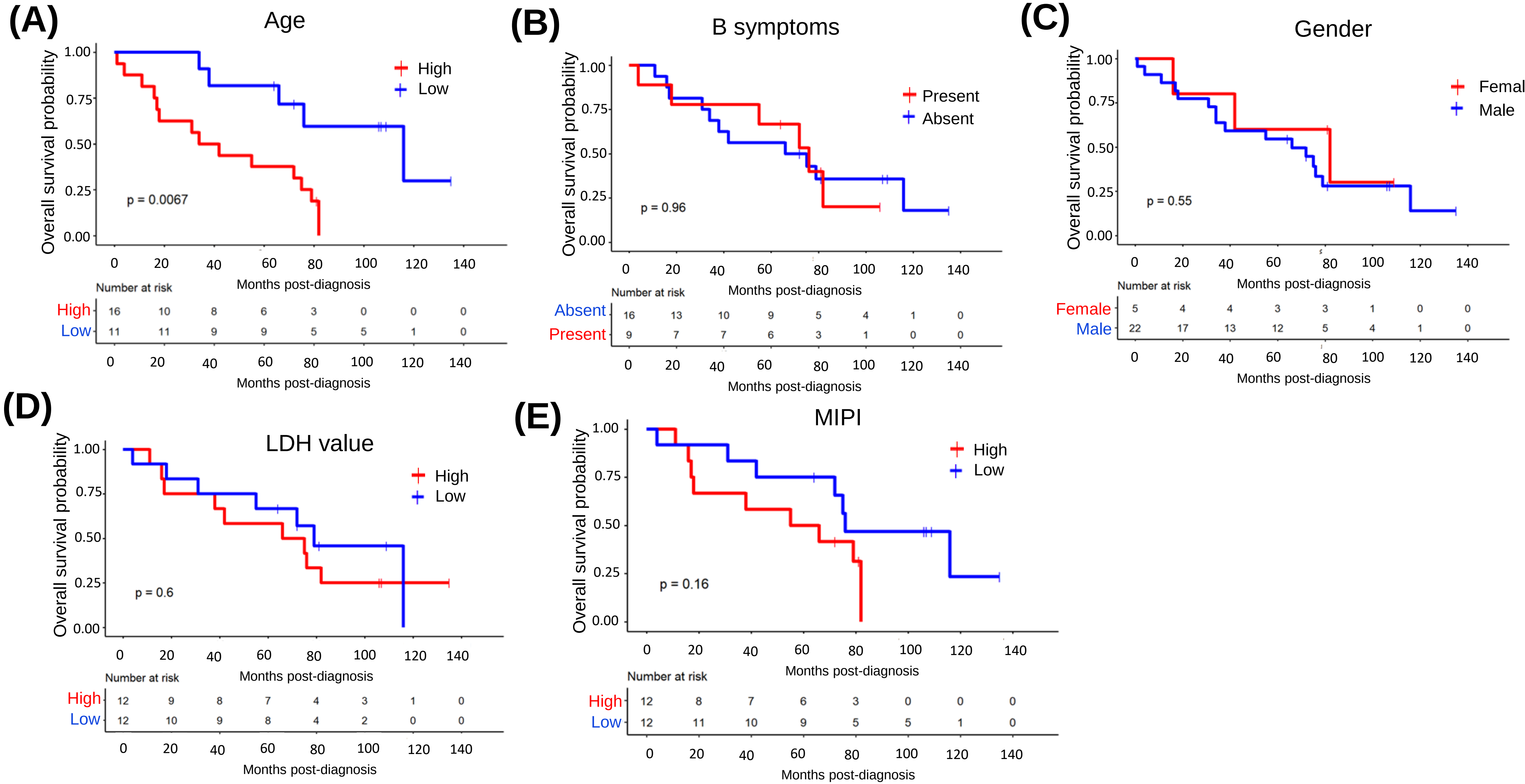

Figure S3. The relationship between clinicopathological, demographic variables and MCL survival. Kaplan Meier curves of diagnostic MCL cases dichotomized based on age (**A**), B symptoms (**B**), gender (**C**), serum LDH levels (**D**), MIPI (**E**). The numbers in parenthesis show the number of MCL patients in each category for different variables.

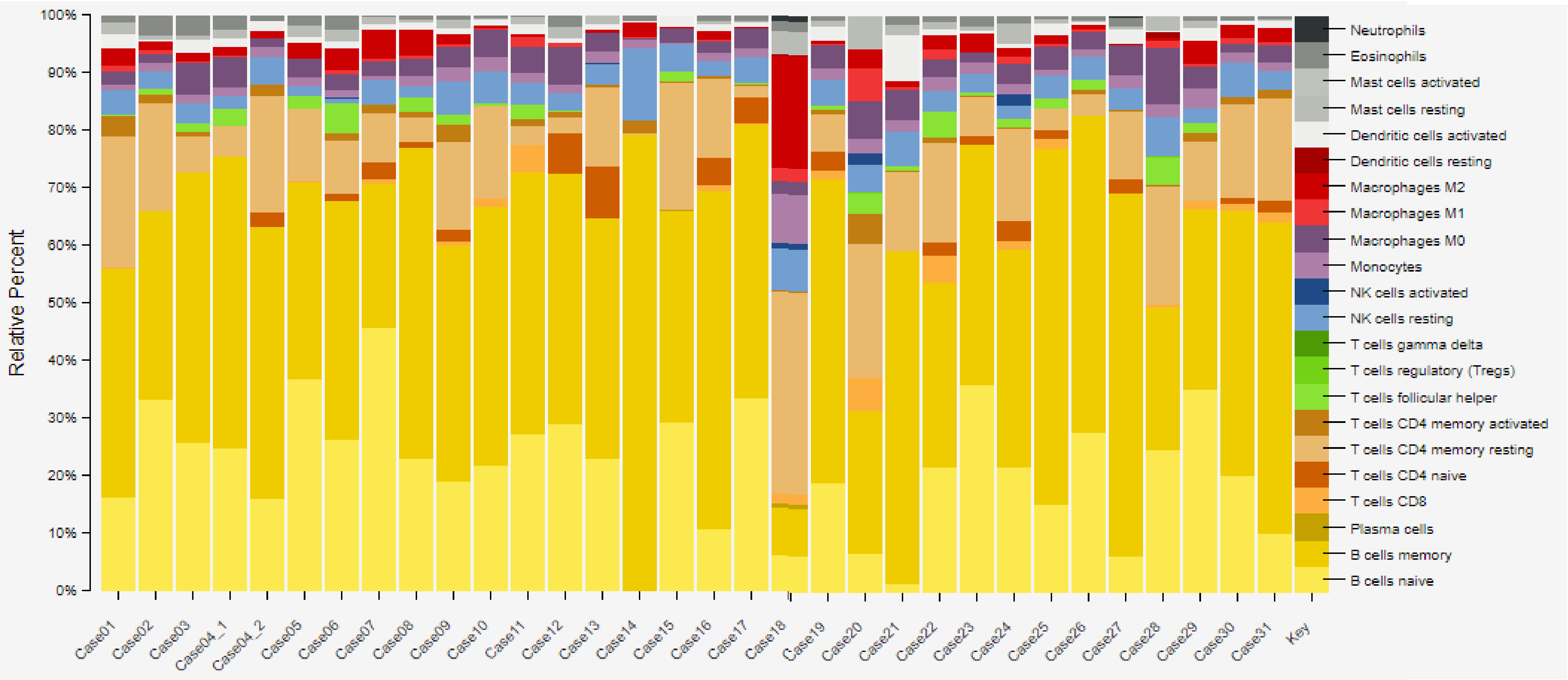

Figure S4. The microenvironmental immunocyte composition of MCL cases. Relative percentage of each of 22 immunocyte predicted with CIBERSORTx based on mRNA expression levels determined with whole transcriptome sequencing of diagnostic or relapsed MCL tumor samples. Each column represents the immune cell percentages in the microenvironment of individual MCL cases.

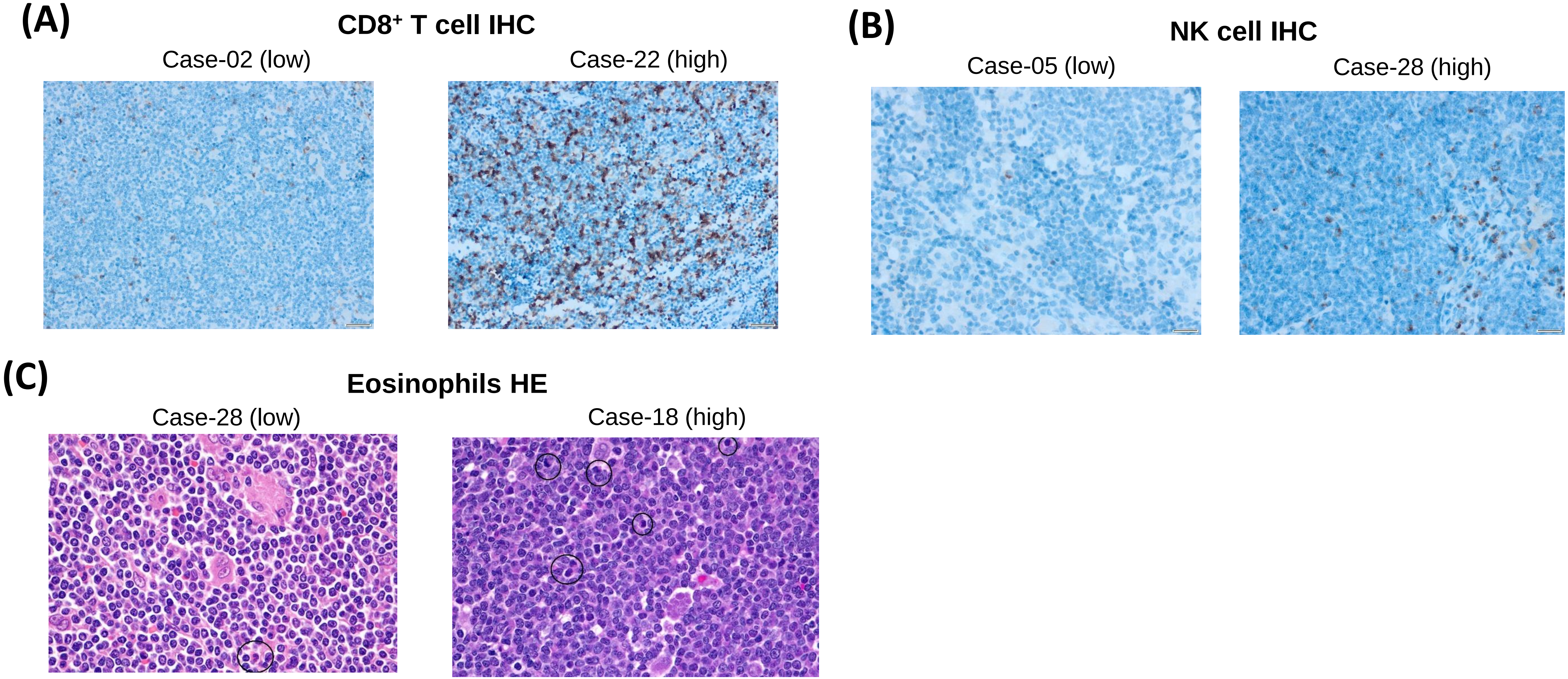

Figure S5. IHC or HE stainings confirm CIBERSORTx estimates in MCL cases. Immunohistochemical staining (IHC) results of tumor tissue sections of representative MCL cases showing low (left panel) or high (right panel) ratio of tumor infiltrating $CD8^+$ T cells (**A**) or NK cells (**B**). IHC stained cells are brown colored. **C**) Hematoxylin-eosin staining (HE) results of tumor sections representing MCL cases with low (left panel) or high (right panel) ratio of tumor infiltrating eosinophils. Eosinophils are shown in black circles. Two hundred X magnification was used for $CD8^+$ T cell IHC images whereas NK cell IHC or eosinophil HE sections were show 400X magnifications. Over each micrograph the MCL case code as well as the CIBERSORTx estimates of cells are indicated.

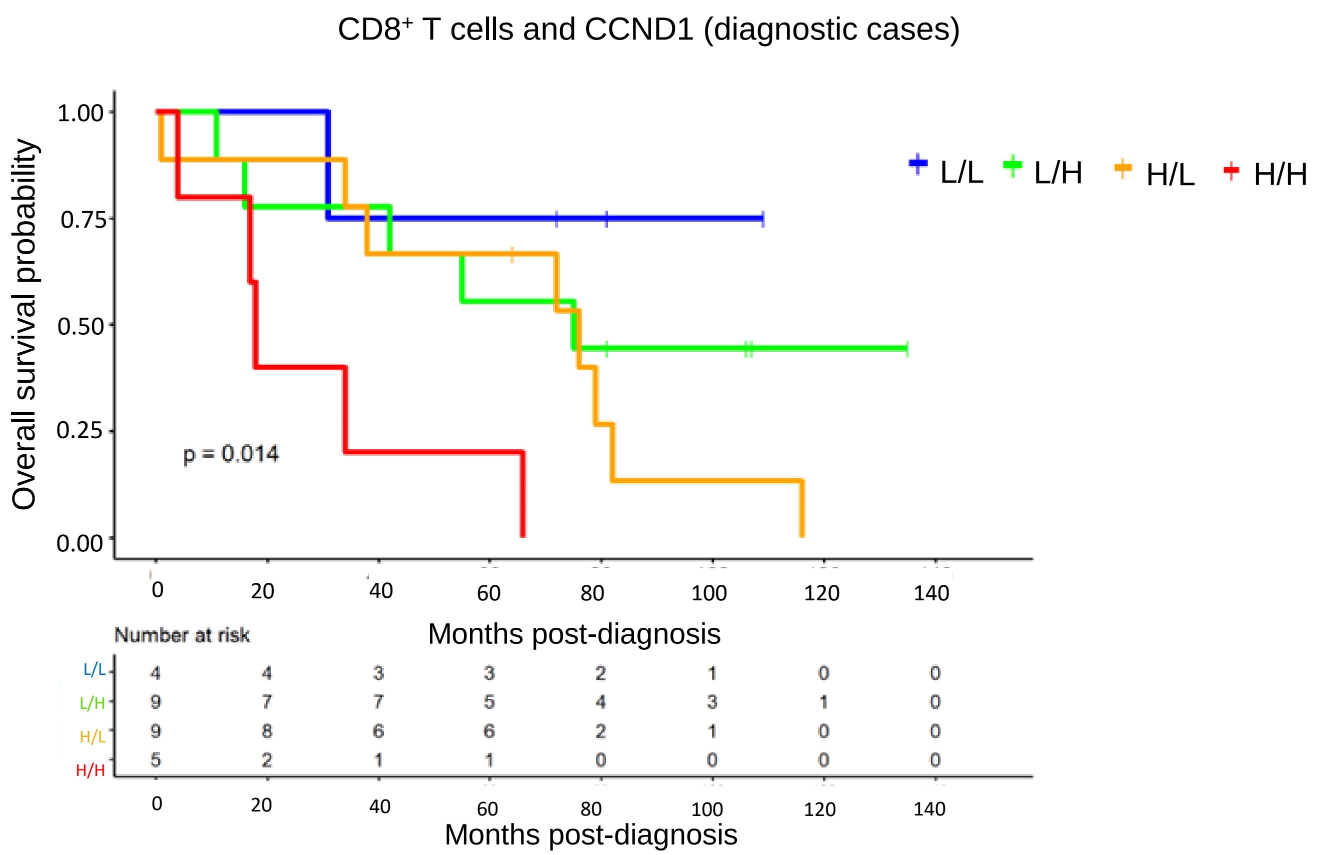

Figure S6. High risk MCL patients with a high ratio of CD8+ T cells and high CCND1 transcript expression levels. Kaplan Meier plot showing the curve based on a combination of CD8+ T cell ratios and CCND1 transcript. L/L: Low CD8+ T cell abundance and low CCND1 expression; L/H: Low CD8+ T cell abundance and high CCND1 expression; H/L: High CD8+ T cell abundance and low CCND1 expression; H/H: High CD8+ T cell abundance and high CCND1 expression.

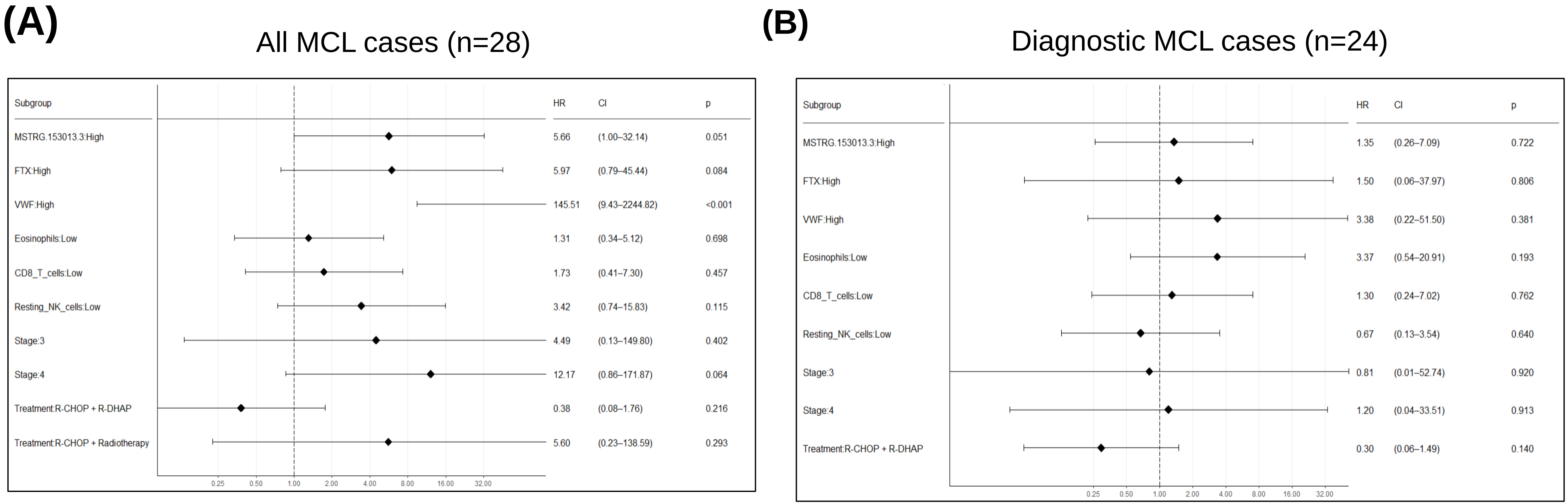

Figure S7. Forest plots of multivariate Cox's regression analysis for overall survival. The forest plots representing the relationship between transcript expression, TME immunocyte composition, disease stage and treatment representing all (**A**) or only diagnostic (**B**) MCL cases. Three of the diagnostic MCL cases (Case-07, Case-11, and Case-19) were not included in the multivariate Cox's regression analyses due to missing data of one or more variables. The treatments indicated are the initial line of treatment of MCL patients.
