# Supplementary material for: Whole Transcriptome Sequencing Reveals Cancer-Related, Prognostically Significant Transcripts and Tumor-Infiltrating Immunocytes in Mantle Cell Lymphoma
Source: Cells. 2022 Oct 27;11(21):3394. doi: 10.3390/cells11213394 (PMC9654955; doi:10.3390/cells11213394)
Supplement: Supplementary file 1 [file cells-11-03394-s001.zip › 28.10.2022-cells-1956495-SI Tables-MCL WTS-MDPI_Cells.pdf]

Table S1. The summary of demographic and clinicopathological characteristics of the MCL cases.

	Overall (N=31)
**Sex**	
Female	5 (16.1%)
Male	26 (83.9%)
**Age (years)**	
Mean (SD)	63.0 (11.4)
Median [Min, Max]	63.0 [39.0, 81.0]
**Stage**	
II	1 (3.2%)
III	3 (9.7%)
IV	25 (80.6%)
NA	2 (6.5%)
**Ki67 Index (%)**	
Mean (SD)	37.4 (18.5)
Median [Min, Max]	30.0 [10.0, 75.0]
NA	2 (6.5%)
**LDH Level (U/l)**	
Mean (SD)	222 (67.5)
Median [Min, Max]	207 [107, 386]
NA	4 (12.9%)
**B symptoms**	
Absent	20 (64.5%)
Present	9 (29.0%)
NA	2 (6.5%)
**Initial Treatment**	
R-CHOP	16 (51.6%)
R-CHOP + R-DHAP	11 (35.5%)
R-CHOP + Radiotherapy	1 (3.2%)
NA	3 (9.7%)
**MIPI**	
High risk	27 (87.1%)
Intermediate risk	1 (3.2%)
Low risk	1 (3.2%)
NA	2 (6.5%)

LDH: Lactate dehydrogenase
R-CHOP: Rituximab- Cyclophosphamide, Hydroxydaunomycin, Oncovin, Prednisone
R-DHAP: Rituximab- Dexamethasone, Cytarabine, Cisplatin
MIPI: Mantle Cell Lymphoma International Prognostic Index
NA: Not available

Table S2. RIN and DV200 scores of MCL and control samples.

<table>
<tr><th>Case no</th><th>WTS sample code</th><th>RIN score</th><th>DV200 (%) score</th><th>Genome sequencing company</th></tr>
<tr><td>Case-01</td><td>Case-01</td><td>2.8</td><td>-</td><td>Novogene</td></tr>
<tr><td>Case-02</td><td>Case-02</td><td>3.2</td><td>-</td><td>Novogene</td></tr>
<tr><td>Case-03</td><td>Case-03</td><td>2.3</td><td>70.34</td><td>Macrogen</td></tr>
<tr><td>Case-04-1</td><td>Case-04-1</td><td>2.4</td><td>-</td><td>Novogene</td></tr>
<tr><td>Case-04-2</td><td>Case-04-2</td><td>2.3</td><td>-</td><td>Novogene</td></tr>
<tr><td>Case-05</td><td>Case-05</td><td>3.3</td><td>80.73</td><td>Macrogen</td></tr>
<tr><td>Case-06</td><td>Case-06</td><td>2.4</td><td>-</td><td>Novogene</td></tr>
<tr><td>Case-07</td><td>Case-07</td><td>2.7</td><td>75.85</td><td>Macrogen</td></tr>
<tr><td>Case-08</td><td>Case-08</td><td>2.2</td><td>71.44</td><td>Macrogen</td></tr>
<tr><td>Case-09</td><td>Case-09</td><td>3.2</td><td>71.31</td><td>Macrogen</td></tr>
<tr><td>Case-10</td><td>Case-10</td><td>1.9</td><td>78.67</td><td>Macrogen</td></tr>
<tr><td>Case-11</td><td>Case-11</td><td>3.1</td><td>72.71</td><td>Macrogen</td></tr>
<tr><td>Case-12</td><td>Case-12</td><td>2.3</td><td>-</td><td>Novogene</td></tr>
<tr><td>Case-13</td><td>Case-13</td><td>2.3</td><td>-</td><td>Novogene</td></tr>
<tr><td>Case-14</td><td>Case-14</td><td>2.3</td><td>-</td><td>Novogene</td></tr>
<tr><td>Case-15</td><td>Case-15</td><td>1.8</td><td>-</td><td>Novogene</td></tr>
<tr><td>Case-16</td><td>Case-16</td><td>2.3</td><td>71.27</td><td>Macrogen</td></tr>
<tr><td>Case-17</td><td>Case-17</td><td>1.7</td><td>-</td><td>Novogene</td></tr>
<tr><td>Case-18</td><td>Case-18</td><td>2.1</td><td>58.26</td><td>Macrogen</td></tr>
<tr><td>Case-19</td><td>Case-19</td><td>2.4</td><td>-</td><td>Novogene</td></tr>
<tr><td>Case-20</td><td>Case-20</td><td>2.3</td><td>-</td><td>Novogene</td></tr>
<tr><td>Case-21</td><td>Case-21</td><td>2.4</td><td>-</td><td>Novogene</td></tr>
<tr><td>Case-22</td><td>Case-22</td><td>1.9</td><td>58.34</td><td>Macrogen</td></tr>
<tr><td>Case-23</td><td>Case-23</td><td>2.4</td><td>-</td><td>Novogene</td></tr>
<tr><td>Case-24</td><td>Case-24</td><td>2.5</td><td>-</td><td>Novogene</td></tr>
<tr><td>Case-25</td><td>Case-25</td><td>2.2</td><td>-</td><td>Novogene</td></tr>
<tr><td>Case-26</td><td>Case-26</td><td>2.6</td><td>-</td><td>Novogene</td></tr>
<tr><td>Case-27</td><td>Case-27</td><td>2.3</td><td>-</td><td>Novogene</td></tr>
<tr><td>Case-28</td><td>Case-28</td><td>2.3</td><td>-</td><td>Novogene</td></tr>
<tr><td>Case-29</td><td>Case-29</td><td>2.5</td><td>-</td><td>Novogene</td></tr>
<tr><td>Case-30</td><td>Case-30</td><td>2.7</td><td>-</td><td>Novogene</td></tr>
<tr><td>Case-31</td><td>Case-31</td><td>2.3</td><td>-</td><td>Novogene</td></tr>
<tr><td>NBC-01</td><td>Control-01</td><td>7.5</td><td>-</td><td>Novogene</td></tr>
<tr><td>NBC-02</td><td rowspan="4">Control-02</td><td>8.8</td><td>-</td><td>Novogene</td></tr>
<tr><td>NBC-03</td><td>5.9</td><td>-</td><td>Novogene</td></tr>
<tr><td>NBC-04</td><td>4.6</td><td>-</td><td>Novogene</td></tr>
<tr><td>NBC-05</td><td>2.7</td><td>-</td><td>Novogene</td></tr>
<tr><td>MBC-01</td><td rowspan="3">Control-03</td><td>8.9</td><td>-</td><td>Novogene</td></tr>
<tr><td>MBC-02</td><td>7.7</td><td>-</td><td>Novogene</td></tr>
<tr><td>MBC-03</td><td>7.9</td><td>-</td><td>Novogene</td></tr>
<tr><td>CC-01</td><td>Control-04</td><td>8.2</td><td>-</td><td>Novogene</td></tr>
<tr><td>CC-02</td><td rowspan="3">Control-05</td><td>8.7</td><td>-</td><td>Novogene</td></tr>
<tr><td>CC-03</td><td>7</td><td>-</td><td>Novogene</td></tr>
<tr><td>CC-04</td><td>5.8</td><td>-</td><td>Novogene</td></tr>
</table>

-: RNA samples **without DV200 (%) score.**

Table S3. Control group samples used in WTS and qRT-PCR validation.

Reactive tonsil code*	WTS sample code	Control group samples	WTS usage (Pooled/non-pooled)	qRT-PCR Validation (SNHG5 or CCND1)
RTT-01	Control-01	NBC-01	Non-pooled	SNHG5
RTT-02	Control-02	NBC-02	Pooled	SNHG5
RTT-03		NBC-03		-
RTT-04		NBC-04		CCND1
RTT-05		NBC-05		CCND1
RTT-06	Control-03	MBC-01	Pooled	SNHG5
RTT-01		MBC-02		CCND1
RTT-07		MBC-03		-
RTT-01	Control-04	CC-01	Non-pooled	-
RTT-03	Control-05	CC-02	Pooled	SNHG5
RTT-04		CC-03		CCND1
RTT-05		CC-04		SNHG5, CCND1
RTT-03	Not used in WTS	MBC-04	Not used	CCND1

-: Control samples **not used** for the **qRT-PCR validation.**
NBC: Reactive tonsil naive B cells
MBC: Reactive tonsil memory B cells
CC: Reactive tonsil centrocytes
RTT: Reactive tonsil tissue
*Each of the reactive tonsil tissues was obtained from a different individual.

Table S4. Basic statistics of whole transcriptome sequencing.

Case number	Sample type	Total raw read number	Q20 (%)	Q30 (%)	GC (%)	Genome sequencing company
Case-01	FFPE tRNA	90831962	97.45	93.86	37.26	Novogene
Case-02	FFPE tRNA	117591776	97.56	94.02	38.75	Novogene
Case-03	FFPE tRNA	82136132	98.704	96.398	43.442	Macrogen
Case-04-1	FFPE tRNA	104570182	96.65	91.20	43.20	Novogene
Case-04-2	FFPE tRNA	96138846	97.47	93.83	40.14	Novogene
Case-05	FFPE tRNA	75192548	98.576	96.125	42.894	Macrogen
Case-06	FFPE tRNA	85592928	97.65	94.22	39.32	Novogene
Case-07	FFPE tRNA	70226712	98.628	96.217	43.538	Macrogen
Case-08	FFPE tRNA	69978056	98.575	96.132	42.519	Macrogen
Case-09	FFPE tRNA	74057924	98.628	96.165	45.730	Macrogen
Case-10	FFPE tRNA	56753632	98.59	96.25	44.27	Macrogen
Case-11	FFPE tRNA	72244348	98.669	96.318	43.420	Macrogen
Case-12	FFPE tRNA	100083980	96.72	91.53	44.74	Novogene
Case-13	FFPE tRNA	106195216	96.73	91.43	43.37	Novogene
Case-14	FFPE tRNA	111891342	97.31	92.7	55.7	Novogene
Case-15	FFPE tRNA	108799404	97.25	92.56	54.71	Novogene
Case-16	FFPE tRNA	63634496	98.86	96.87	42.98	Macrogen
Case-17	FFPE tRNA	100157812	96.52	91.11	45.82	Novogene
Case-18	FFPE tRNA	75514478	98.673	96.051	56.539	Macrogen
Case-19	FFPE tRNA	97789426	96.79	91.6	43.69	Novogene
Case-20	FFPE tRNA	108569252	96.52	91.2	46.2	Novogene
Case-21	FFPE tRNA	106357030	96.56	91.32	45.03	Novogene
Case-22	FFPE tRNA	68515654	98.643	96.234	44.568	Macrogen
Case-23	FFPE tRNA	103598684	96.43	90.81	42.42	Novogene
Case-24	FFPE tRNA	112811778	97.31	93.4	46.84	Novogene
Case-25	FFPE tRNA	106341400	96.68	91.39	43.39	Novogene
Case-26	FFPE tRNA	92950872	97.39	93.65	41.43	Novogene
Case-27	FFPE tRNA	88991804	97.48	93.8	41.97	Novogene
Case-28	FFPE tRNA	101516630	95.97	89.94	42.43	Novogene
Case-29	FFPE tRNA	103480146	96.56	91.13	42.7	Novogene
Case-30	FFPE tRNA	104468972	96.59	91.19	43.13	Novogene
Case-31	FFPE tRNA	81421512	97.33	93.53	40.75	Novogene
Control-01	NBC RNA	101290336	97.13	92.95	44.98	Novogene
Control-02	NBC RNA (4 control samples pooled)	104674474	97.11	92.78	51.35	Novogene
Control-03	MBC RNA (3 control samples pooled)	105511034	96.84	92.48	56.62	Novogene
Control-04	CC RNA	103685958	97.31	93.27	46.25	Novogene
Control-05	CC RNA (3 control samples pooled)	108084430	97.19	92.87	50.58	Novogene

FFPE tRNA: Formalin-fixed paraffin-embedded tumor tissue RNA
NBC RNA: Naive B cells RNA
MBC RNA: Memory B cells RNA
CC RNA: Centrocytes RNA
GC(%): GC content.
Q20(%): Ratio of bases that have phred quality score of over 20.
Q30(%): Ratio of bases that have phred quality score of over 30.
